# Mechanically Assisted Magnetic Actuation in Ceramic‐Based Microscrolls for Fast and Durable Soft Robotic Systems

**DOI:** 10.1002/adma.74544

**Published:** 2026-08-08

**Authors:** Semi Kim, Shravan R. Kousik, Petia Atanasova, Eberhard Goering, Joachim Bill, Zaklina Burghard

**Affiliations:** ^1^ Institute for Materials Science University of Stuttgart Stuttgart Germany; ^2^ Solid State Spectroscopy Department Max Planck Institute for Solid State Research Stuttgart Germany

**Keywords:** ceramic‐based films, magnetic actuation, microscrolls, soft robotics, stimuli‐responsive materials, vanadium pentoxide

## Abstract

Soft magnetic actuators capable of fast, remote, and untethered motion are increasingly sought for microscale robotic systems. Here, we introduce compact ceramic‐based, magnetically responsive microscroll actuators inspired by the coiled geometry of the butterfly proboscis. The actuators are fabricated from hybrid films composed of aligned vanadium pentoxide (V_2_O_5_) nanofibers and Fe_3_O_4_ nanoparticles distributed within the nanofiber matrix, forming a flexible, laminated architecture with enhanced mechanical robustness. Using a razor blade‐assisted scrolling method, the planar films are transformed into tightly wound microscrolls with tunable geometry and micrometer scale diameters. Under near‐field magnetic stimulation (∼60 mT), the scrolls exhibit rapid, reversible, and multidirectional actuation with angular displacements of up to 180°. The actuation relies on a dual magneto‐mechanical mechanism: distributed magnetic stresses generated by the embedded Fe_3_O_4_ phase initiate unrolling, while residual elastic strain stored during scrolling drives the re‐rolling motion. This geometry‐programmed actuation enables a lifting ratio of 32.5× relative to actuator mass, a work density of ∼8.1 kJm^−^^3^, and a footprint reduction of up to 96%. Notably, the ceramic‐based microscrolls retain structural and functional integrity over 5000 magnetic actuation cycles, demonstrating a durable architecture‐driven route toward untethered soft robotic microsystems.

## Graphical abstract

Ceramic‐based V_2_O_5_/Fe_3_O_4_ hybrid films are shaped into butterfly‐proboscis‐inspired microscroll actuators. Magnetic triggering initiates unrolling, while geometry‐programmed elastic strain energy drives rapid recoil, enabling fast, reversible, durable, and load‐bearing actuation under near‐field magnetic stimulation. With up to 96% footprint reduction and a lifting ratio of 32.5× the actuator mass, these microscrolls support wireless gripping, lifting, and switching, offering a versatile platform for compact soft robotic and micromechanical systems.

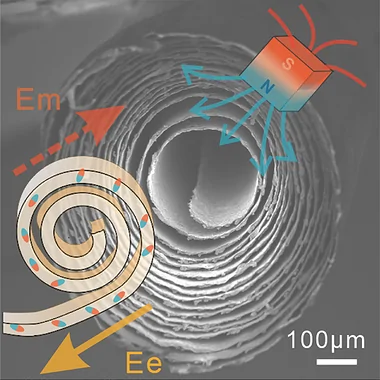

## 1 Introduction

Soft robotic systems increasingly rely on compliant, stimuli‐responsive materials that mimic the adaptive and multifunctional motions of biological structures [1, 2, 3, 4]. Extensive research has explored shape‐memory polymers [5], liquid crystal elastomers [6], hydrogels [7], and other soft matter platforms for achieving programmable motion, gripping, and locomotion. These materials enable reconfigurable actuators capable of complex deformation pathways while maintaining mechanical compliance [8]. A continuing challenge, however, lies in developing soft materials that combine rapid response, structural robustness, and scalable fabrication in architectures suited for micro‐robotic and untethered operation [9, 10]. Recent reviews further emphasize that future soft actuator systems require improved scalability, reproducibility, mechanical durability, and integration into untethered robotic platforms operating under realistic environmental conditions [11].

Among external stimuli used to actuate soft systems [12], magnetic fields stand out due to their ability to enable fast, wireless [13], and precisely controlled actuation even through barriers and without physical contact [14]. Magnetically responsive composites, typically made by dispersing magnetic fillers into elastomers or polymers, have been widely investigated [15, 16]. Although effective, these systems often require high filler loadings (20–70 wt%) and millimeter‐scale thicknesses to achieve sufficient magnetic torque, which compromises flexibility and cycling durability [17, 18, 19]. To overcome these limitations, alternative lightweight and compliant architectures have been explored [20]. For instance, Fe_3_O_4_‐impregnated cellulose‐based actuators have shown good deformation response and cycle stability, although still limited by relatively low output forces and actuation thresholds around 90 mT [21]. Recent advances have demonstrated ultrafast magnetic soft robots capable of operating at exceptionally low fields (0.5–2 mT), achieving high‐frequency actuation (up to 200 Hz) and specific energy densities exceeding 10 kJ m^−^^3^ mT^−^^1^, highlighting the potential of optimized magnetoelastic architectures [22]. Recent efforts have additionally focused on reprogrammable magnetic soft actuators and fiber‐based magnetic robotic systems capable of complex, multimodal deformation under remote magnetic control [23, 24, 25]. By fabricating lightweight, micrometer‐thick films with high mechanical compliance, it becomes possible to achieve significant deformation under relatively weak magnetic fields [26], yet further improvement requires architectures that integrate miniaturization, durability, and efficient magnetic response.

Bioinspired strategies represent a promising route to meet these needs, by drawing inspiration from compact, efficient, and reversible motion mechanisms found in nature [27]. Natural structures such as the butterfly proboscis exhibit compact, reversible uncoiling driven by their intrinsic multilayered geometry [28]. Several proboscis‐inspired actuators have been developed, including rolled liquid crystal networks and spiral structures combining polymer tubes with dielectric fluid actuators. Sol et al. developed tightly rolled, tapered actuators based on 25 µm‐thick liquid crystal networks, demonstrating multiple windings in response to heat (80 °C) or blue light (455 nm). These actuators offered smooth, reversible transitions between rolled and unrolled states, though their long response times (∼40 min) limit their utility in dynamic applications [29]. Another example is the work by Lin et al., who fabricated a spiral actuator by coupling a low‐density polyethylene tube with a dielectric fluid transducer pouch to replicate butterfly proboscis motion [30]. Despite its conceptual elegance and reversible uncoiling motions, the system remains bulky (millimeter range) and involves a multi‐component architecture, which limits its applicability in miniaturized or integrated soft robotic systems.

Ceramic‐based thin films represent an alternative material platform capable of addressing these challenges in actuator miniaturization, structural robustness, and low‐field responsiveness. Vanadium pentoxide nanofiber (VNF) films, formed through the self‐assembly of high‐aspect‐ratio nanofibers, exhibit layered architectures, hydrogen‐bond‐mediated cohesion, and Young's moduli near 30 GPa comparable to structural polymers [31]. The films maintain microscale flexibility and allow integration of functional nanomaterials such as graphene oxide or cellulose nanofibers without compromising mechanical integrity [32, 33]. These features make VNF films promising candidates for compact, mechanically robust, and magnetically responsive actuators.

Here, we introduce ceramic‐based microscroll actuators fabricated from hybrid VNF/Fe_3_O_4_ films that combine compact geometry, multidirectional magnetic responsiveness, and long‐term durability. The hybrid films are prepared through a scalable self‐assembly route, in which 8.6–11 wt% Fe_3_O_4_ nanoparticles are incorporated into an aligned V_2_O_5_ nanofiber matrix to form freestanding hybrid composite films. From these planar hybrid films, scroll‐like structures are fabricated using a razor blade assisted method, reminiscent of the coiled morphology of the butterfly proboscis. Systematic characterization reveals that the scrolling process stores residual elastic strain energy within the multilamellar film structure, which together with distributed magnetic stresses generated by the Fe_3_O_4_ phase give rise to a dual magneto‐mechanical actuation mechanism. This mechanism enables magnetically triggered unrolling and mechanically assisted recoil, establishing the foundation for high‐performance magneto‐mechanical response explored in this study. In this context, the present multilamellar microscroll architecture contributes to emerging efforts toward compact, array‐compatible, and remotely addressable magnetic actuator systems [34]. While magnetically induced deformation combined with elastic recoil has been widely explored in magnetic soft actuator systems [35] and programmable soft robotic architectures [10, 13], the present work focuses on a ceramic‐based multilamellar microscroll architecture, in which actuation behavior is governed by geometry‐programmed elastic energy storage. In contrast to conventional soft magnetic actuators, which typically rely on soft, low‐stiffness materials, the combination of high‐modulus inorganic nanofiber films and scroll geometry enables enhanced load‐bearing capability at the microscale, rapid and repeatable recoil driven by stored elastic strain, and compact‐to‐expanded reversible configurations.

## 2 Results and Discussions

### 2.1 Fabrication of VNF/Fe_3_O_4_ Hybrid Film

**FIGURE 1:**
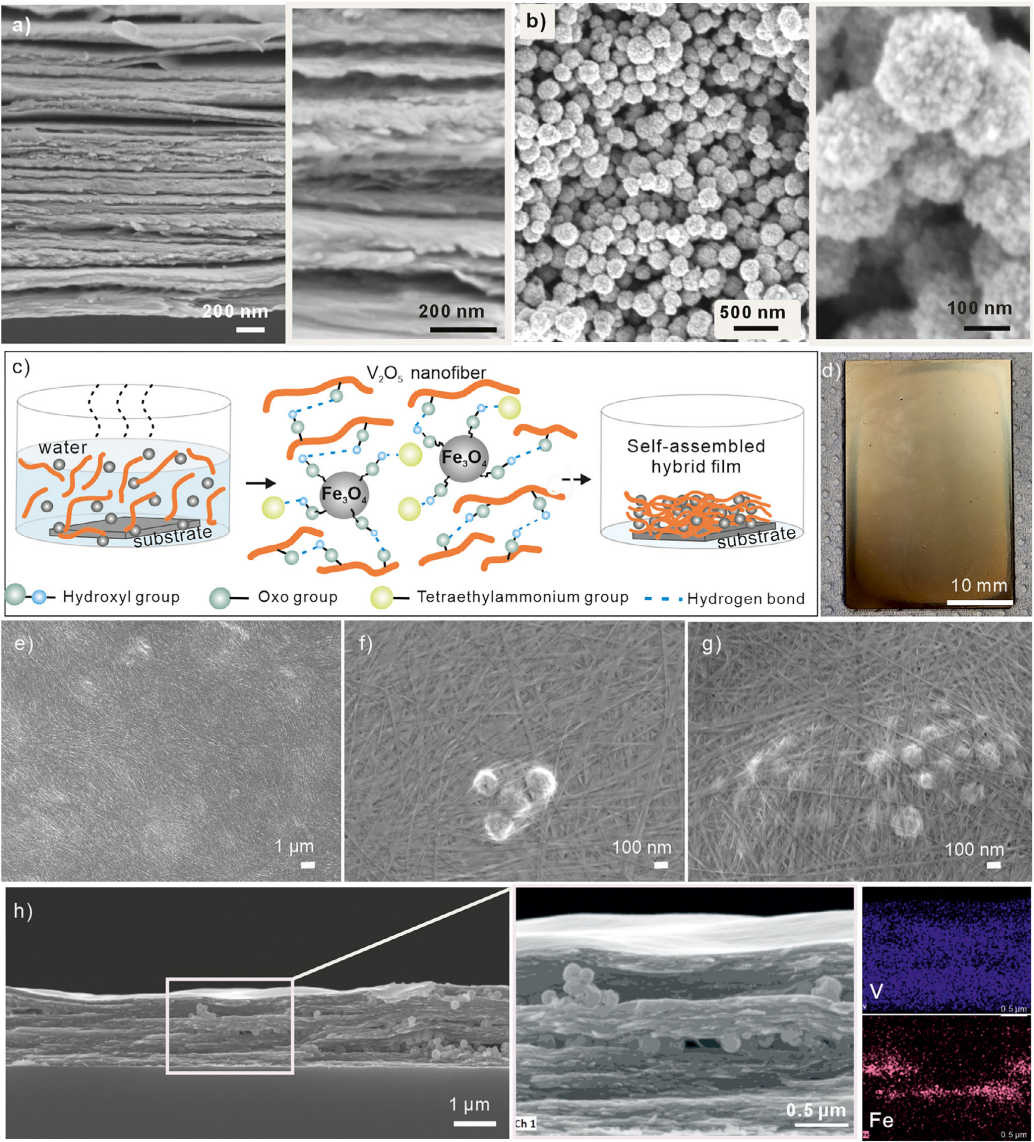
Fabrication and structural characterization of VNF/Fe_3_O_4_ hybrid films. (a) Cross‐sectional SEM images of a self‐assembled VNF film at two magnifications, showing its layered nanofiber architecture. (b) SEM images of Fe_3_O_4_ nanoparticles revealing spherical hierarchical secondary particles. Higher magnification shows the textured surface morphology of individual particles, consistent with their nanoscale substructure. (c) Schematic illustration of the self‐assembly process used to fabricate hybrid films. (d) Digital image of the as‐prepared hybrid film cast on a glass substrate. (e) SEM images of the VNF/Fe_3_O_4_ hybrid film showing Fe_3_O_4_ particle‐rich regions distributed on the film surface. (f and g) High‐magnification SEM image showing Fe_3_O_4_ particles embedded and locally covered by the VNF nanofiber network. (h) Cross‐sectional SEM image of a hybrid film containing 8.6 wt% Fe_3_O_4_ showing the multilayered VNF with embedded spherical Fe_3_O_4_ particles. Corresponding EDX elemental maps display vanadium (V, blue) associated with VNF lamellae and iron (Fe, pink) originating from Fe_3_O_4_ nanoparticles.

Vanadium pentoxide nanofibers (VNFs) were synthesized via a heat‐induced sol–gel polycondensation process of ammonium metavanadate in aqueous solution [36]. By adjusting the sol aging time, the fiber length can be tuned [37]. In this study, the average length was ∼2 µm. The presence of surface functional groups, such as hydroxyl (─OH) and carbonyl (═O) moieties, enables these nanofibers to self‐assemble from aqueous dispersions into layered, paper‐like films several micrometers thick. A typical cross‐sectional SEM image of such obtained film is presented in Figure 1a. Higher magnification reveals densely packed and well‐aligned nanofibers in the spatial direction, forming a highly ordered layered architecture. A network of hydrogen bonds promotes parallel stacking of the nanofibers, leading to a compact and anisotropic structure. Due to their surface functional groups, the fibers carry a negative charge and exhibit versatile chemical functionality, allowing incorporation of additional functional components [35]. To introduce magnetic responsiveness, Fe_3_O_4_ nanoparticles were embedded within the V_2_O_5_ matrix. Among spinel ferrites, Fe_3_O_4_ offers high saturation magnetization, thermal stability, and superparamagnetic behavior at the nanoscale, minimizing remanent magnetization and particle aggregation [38]. Fe_3_O_4_ nanoparticles were synthesized via solvothermal hydrolysis of iron (III) chloride hexahydrate (FeCl_3_·6H_2_O), yielding spherical particles with an average diameter of ∼200 nm (Figure 1b). Closer examination of the microstructure revealed that the spherical Fe_3_O_4_ particles are hierarchical secondary particles composed of aggregated Fe_3_O_4_ nanocrystallites (<30 nm), consistent with their textured surface morphology. For simplicity, these hierarchical secondary particles are referred to as Fe_3_O_4_ nanoparticles throughout the manuscript. To stabilize the aqueous dispersion, tetraethylammonium hydroxide (TEAOH) was added as an electrostatic dispersant [39].

The hybrid films were prepared by mixing the stabilized Fe_3_O_4_ dispersion with VNF sols and casting the mixture onto glass substrates. As water evaporated, the components self‐assembled into hybrid films featuring a layered nanofiber architecture intercalated with Fe_3_O_4_ nanoparticles, as schematically presented in Figure 1c. Hydrogen bonding between the hydroxyl groups on the Fe_3_O_4_ nanoparticles and oxygen‐containing functional groups on the VNFs surface likely facilitates their uniform integration into the lamellar architecture [40]. Controlled casting parameters, including dispersion volume and concentration, yielded golden colored films several micrometers thick with uniform coloration indicating consistent film thickness (Figure 1d).

Comparison with the microstructure of hybrid films containing Fe_3_O_4_ particles without TEAOH (Figure S1, Supporting Information) shows that the use of TEAOH as electrostatic dispersant not only improves colloidal stability of Fe_3_O_4_ nanoparticles but also facilitates their uniform incorporation into the VNF‐layered structure. This results in consistent film thickness and well‐preserved lamellar order. Surface SEM imaging confirmed the interconnected nanofibrous morphology of the V_2_O_5_ matrix and additionally revealed embedded Fe_3_O_4_ particle‐rich domains beneath the surface nanofiber network (Figure 1e–g). Furthermore, a representative fracture‐surface cross‐section of a hybrid film containing 8.6 wt% Fe_3_O_4_, presented in Figure 1h, reveals a ∼4 µm thick multilayered architecture in which spherical particles are embedded between stacked VNF lamellae. Higher‐magnification imaging indicates that nanoparticles are primarily located within interlamellar regions, where they are observed as small particle‐rich domains, consistent with their hierarchical secondary‐particle morphology described above. The layered architecture of the VNF matrix remains intact and continuous, indicating that incorporation of Fe_3_O_4_ does not disrupt the underlying nanofiber alignment. The corresponding EDX elemental maps further verify the distribution of Fe_3_O_4_ nanoparticles within the hybrid film, with vanadium (V) from the VNF matrix uniformly visible across the lamellae and iron (Fe) appearing as dispersed localized signals. These results further confirm that the hybrid films maintain structural integrity while accommodating the magnetic nanoparticles. To identify the optimal magnetic loading, films containing 3–17 wt% Fe_3_O_4_ were prepared and analyzed. Films containing below 8.6 wt% Fe_3_O_4_ exhibited negligible magnetic response, whereas loadings above 11 wt% resulted in film brittleness and partial delamination (Figure S2, Supporting Information). Therefore, compositions with 8.6 and 11 wt% Fe_3_O_4_ were selected for further structural and functional characterization.

### 2.2 Magnetic and Mechanical Properties of VNF/Fe_3_O_4_ Hybrid Film

**FIGURE 2:**
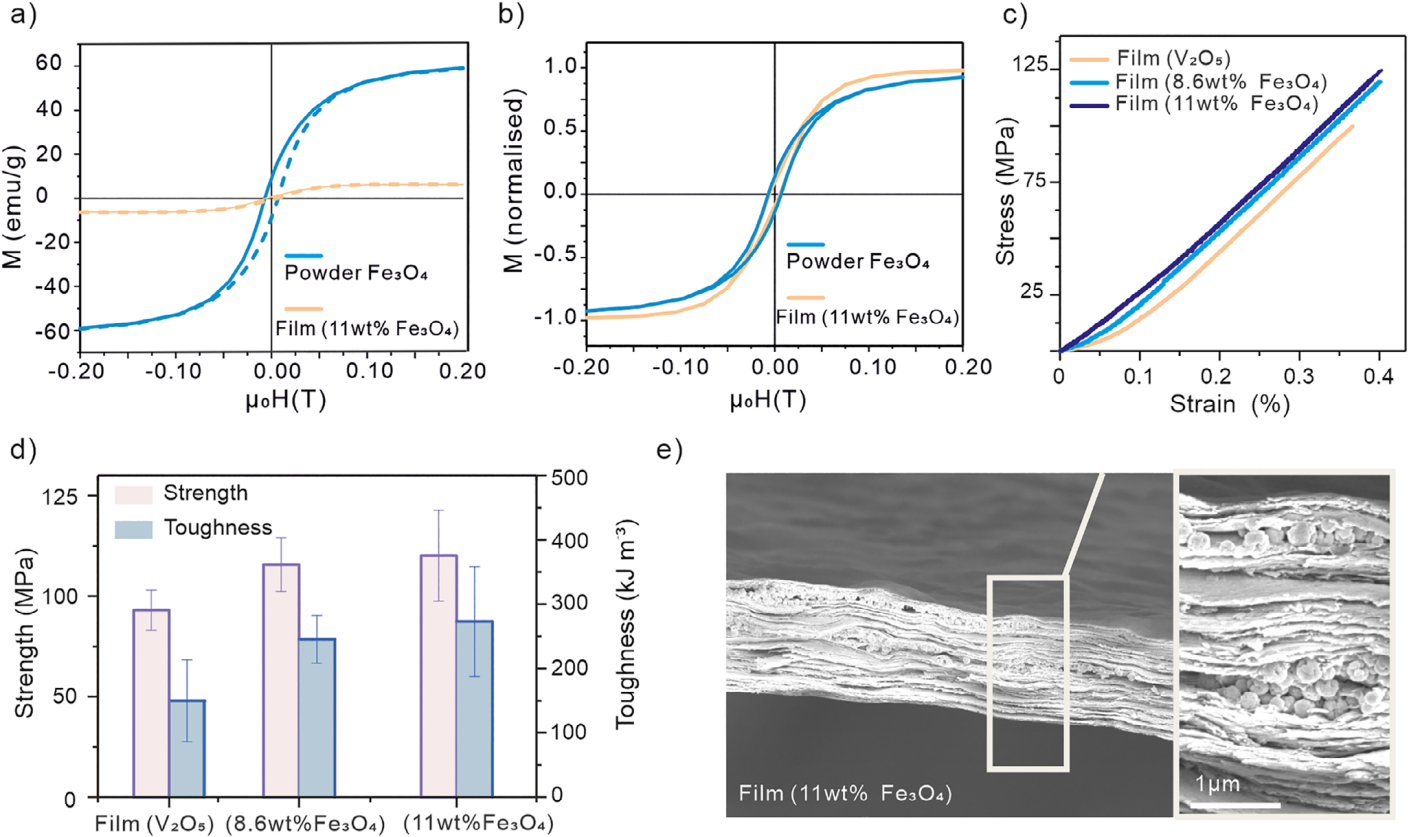
Magnetic and mechanical characterization of Fe_3_O_4_ nanoparticles and VNF/Fe_3_O_4_ hybrid films. (a) Magnetization curves of Fe_3_O_4_ nanoparticles and the VNF/Fe_3_O_4_ hybrid film (11 wt%) as a function of applied magnetic field at room temperature. (b) Normalized magnetization curves (M/M_s_) reveal that the intrinsic magnetic behavior of Fe_3_O_4_ nanoparticles is preserved in the hybrid film. (c) Representative stress–strain curves for pure VNF and hybrid films. (d) Tensile strength and toughness values derived from the corresponding stress–strain measurements. (e) Cross‐sectional SEM images of fractured hybrid films containing 11 wt% Fe_3_O_4_, showing multilayered fracture morphology with nanoparticle‐rich domains embedded within the VNF lamellar structure.

The magnetic properties of VNF/Fe_3_O_4_ hybrid films were characterized using SQUID magnetometry and compared to those of pure Fe_3_O_4_ nanoparticles. The measured magnetization (M) as a function of the applied magnetic field is presented in Figure 2a. The reference Fe_3_O_4_ nanoparticles, exhibited a saturation magnetization of 64.3 emu g^−1^, consistent with values reported for superparamagnetic Fe_3_O_4_ particles [41]. SEM analysis (Figure 1b) reveals that the Fe_3_O_4_ powder consists of spherical hierarchical secondary particles composed of primary nanoparticles in the sub‐30 nm range. This size range lies close to the superparamagnetic blocking threshold for magnetite, which explains the observed small but present coercivity and remanence at room temperature, indicating that a minor fraction of nanoparticles remains weakly blocked.

In contrast, the hybrid film containing 11 wt% Fe_3_O_4_ shows a significantly reduced saturation magnetization of 6.95 emu g^−1^. This reduction reflects both the lower magnetic mass fraction and the increased spacing between Fe_3_O_4_‐rich domains that redisperse into isolated nanoparticles within the VNF matrix. A similar monotonic decrease in saturation magnetization with reduced Fe_3_O_4_ content has been widely reported for magnetic composite systems, including polymer‐based matrices [42], where dilution of the magnetic phase lowers the overall magnetization while individual nanoparticles retain their intrinsic magnetic behavior.

When the magnetization curves are normalized to their respective saturation values (Figure 2b), the hybrid film and pure Fe_3_O_4_ nanoparticles exhibit nearly identical field dependence. This confirms that the intrinsic partially blocked superparamagnetism of Fe_3_O_4_ is preserved within the hybrid matrix and that incorporation into VNF does not chemically or structurally degrade the nanoparticles. Small uncertainties in the effective Fe_3_O_4_ loading, such as incomplete nanoparticle transfer during film casting or residual hydration of the oxide matrix, can introduce systematic errors in mass normalization, explaining part of the difference in absolute saturation magnetization. Importantly, these variations do not influence the magnetic functionality relevant for scroll actuation. A comparable trend was observed in the hybrid film containing 8.6 wt% Fe_3_O_4_, which exhibited slightly lower magnetization, consistent with reduced particle content (Figure S3, Supporting Information). These results indicate that the blocking temperature and magnetic response of the hybrid films are governed primarily by the size of the Fe_3_O_4_ primary nanoparticles, with minimal influence from the VNF matrix. While these static measurements confirm that the Fe_3_O_4_ phase retains its partially blocked superparamagnetic behavior within the hybrid matrix, they do not resolve the relative contributions of field‐induced magnetization, local particle‐level torque, distributed magnetic stresses, or bulk gradient‐driven body forces to the macroscopic actuation response.

The mechanical performance of the hybrid films was evaluated through uniaxial tensile testing and compared to that of the pure VNF film. For comparison, we used mechanical property data of the VNF film obtained in our previous work as a reference to assess the effect of Fe_3_O_4_ nanoparticle incorporation [43]. It is worth noting that all mechanical tests were conducted on free‐standing films. The fact that the hybrid films could be detached from the substrate as intact and manipulated without additional support highlights their mechanical robustness. The stress–strain curves (Figure 2c) display a nearly linear response up to fracture for all compositions, without detectable plastic deformation. The similar slopes of the curves suggest that the incorporation of Fe_3_O_4_ nanoparticles has only a minor influence on the elastic response of the hybrid films. The Young's modulus of the pure VNF film (32 ± 3 GPa), is largely preserved in the hybrid films, with values of 29 ± 4 and 28 ± 5 GPa for the samples containing 8.6 and 11 wt% Fe_3_O_4_, respectively. This behavior can be rationalized by the hybrid film microstructure. The continuous, well‐aligned VNF lamellae form the primary load‐bearing scaffold across the film thickness, whereas the Fe_3_O_4_ phase is present as non‐percolating particles and particle‐rich domains located mainly within interlamellar regions. As a result, the elastic modulus is governed mainly by the VNF lamellar network, while local deviations arise from disruptions in lamellar alignment and cavity‐like features associated with Fe_3_O_4_‐reach domains on the fracture surface (Figure 2e). The hierarchical secondary particle morphology and limited interparticle connectivity further restrict the formation of a continuous particle‐reinforced framework, explaining why Fe_3_O_4_ incorporation does not lead to a pronounced stiffness enhancement.

The tensile strength of the hybrid films shows a moderate, yet clearly observable increase upon nanoparticle incorporation (Figure 2d), rising from 93 ± 10 MPa for the pure VNF film to 116 ± 13 and 120 ± 23 MPa for hybrid films containing 8.6 and 11 wt% Fe_3_O_4_, respectively, representing approximate increases of up to 30%. In contrast, substantial improvement in toughness, by 63% and 82% for 8.6  and 11 wt% Fe_3_O_4_, respectively, supports the presence of dissipative deformation mechanisms during fracture. This behavior is reminiscent of toughening concepts known from nacre‐like materials [44], where hierarchical layered architectures promote crack deflection, interfacial sliding, and energy dissipation [45]. In the present hybrid films, these mechanisms include crack deflection and pinning at Fe_3_O_4_‐rich domaines, interlamellar sliding and partial delamination of VNF lamellea, and frictional energy dissipation associated with interfacial sliding and debonding along VNF–VNF and VNF–Fe_3_O_4_ interfaces [46]. In this context, the hydrogen‐bonded VNF matrix provides numerous weak interfaces that can progressively decohere in the crack‐tip process zone, so that many small interfacial failure events collectively contribute to the increased fracture energy without introducing macroscopic plasticity. A stepwise schematic of the proposed microstructural evolution under tensile loading is provided in Figure S5.

Consistent with this interpretation, cross‐sectional SEM images reveal rough and frayed fracture surfaces in both hybrid films, while the overall lamellar architecture remains preserved after failure (Figures 2e and S4, Supporting Information). The 8.6 wt% film shows a more homogeneous distribution of Fe_3_O_4_‐rich domains within the lamellar network, which may promote more uniform stress redistribution and crack arrest. In the 11 wt% film, larger Fe_3_O_4_‐rich domains and more pronounced cavity‐like features locally increase structural heterogeneity and may act as stress concentrators. However, these features can also enhance crack deflection, interfacial sliding, and pull‐out [45]. This competition between increased flaw sensitivity and enhanced energy dissipation is consistent with the higher toughness and larger scatter in strength observed for the 11 wt% composition.

### 2.3 Fabrication of Magnetic Microscrolls From VNF/Fe_3_O_4_ Hybrid Films

**FIGURE 3:**
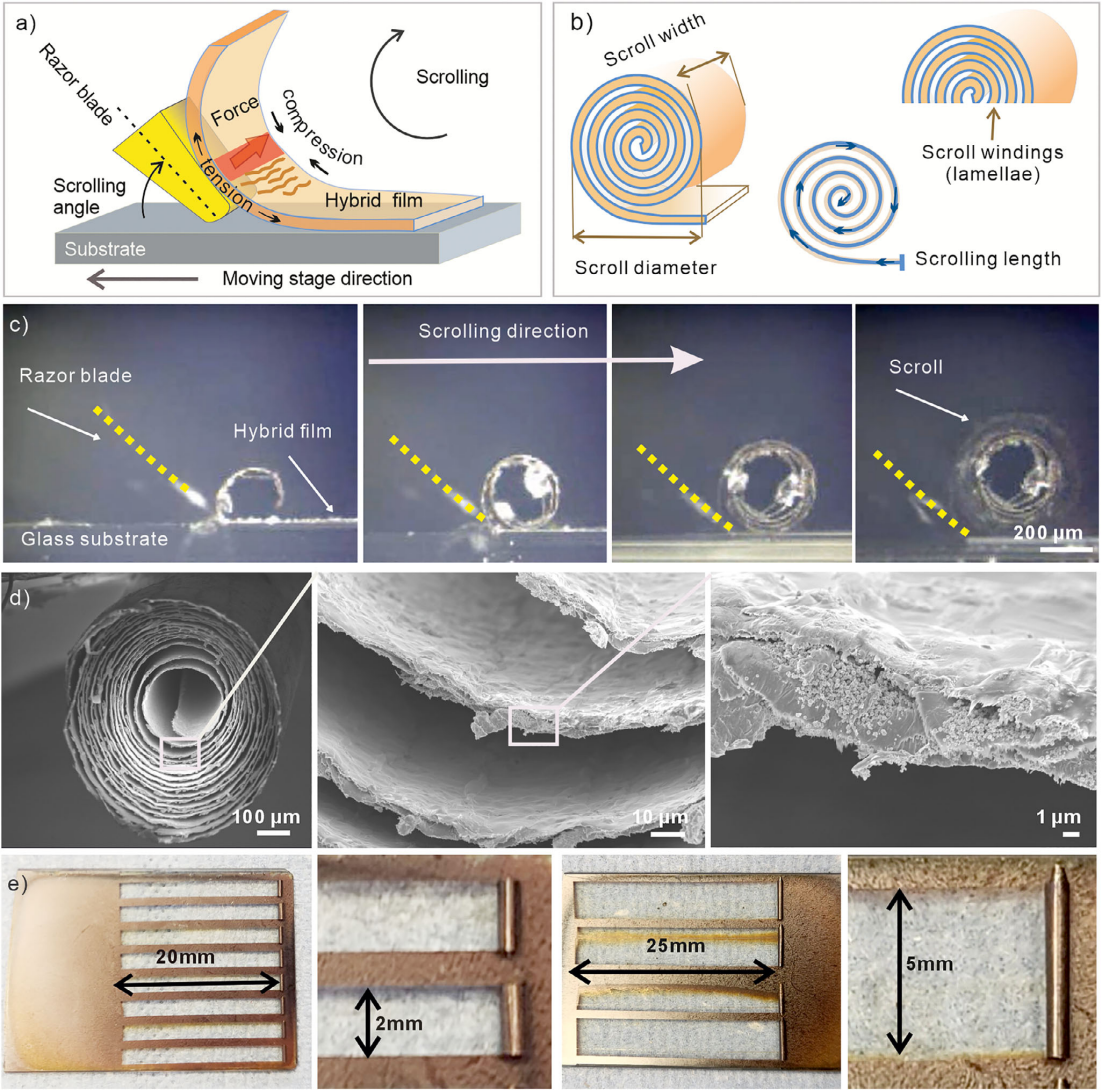
Fabrication and structural architecture of VNF/Fe_3_O_4_ microscrolls. (a) Schematic of scroll fabrication using a razor blade, illustrating the scrolling process. (b) Definition of the key geometric parameters of the scroll. (c) Sequential digital light microscopy images from cross‐sectional recordings demonstrating the scrolling process (Video S1, Supporting Information). (d) Cross‐sectional SEM image of a 2 mm × 25 mm microscroll, with higher‐magnification views showing the internal layered structure. (e) Digital images of VNF/Fe_3_O_4_ microscrolls on glass substrates, with varying scrolling lengths (20 and 25 mm) and widths (2 and 5 mm), respectively.

To transform the VNF/Fe_3_O_4_ hybrid films into compact, magnetically responsive actuators, a razor blade‐assisted scrolling method previously established by our group for flexible nanostructured films was employed [47]. In this process, a razor blade fixed at a 30° angle was brought into contact with the hybrid film supported on a glass substrate mounted on a motorized translation stage (schematically illustrated in Figure 3a). Because the blade geometry, substrate configuration, and contact conditions are controlled, the friction conditions governing the delamination–winding sequence can be reproduced in a controlled and consistent manner.

Upon contact, the blade induces controlled delamination of the film from the substrate, initiating bending and the formation of the first winding of the scroll. As the stage advances at a constant speed of 1 mm s^−^^1^, the delaminated section continues to bend and roll into a stable microscroll. During this bending, the inner side of the film experiences compressive strain, while the outer side is subjected to tension, consistent with classical bending mechanics [48]. The final scroll geometry, (diameter, number of lamellae (windings), and overall compactness) was controlled by adjusting the scrolling length, while the scroll width was defined by the width of the blade (Figure 3b). Sequential light‐microscopy images extracted from cross‐sectional video recordings (Figure 3c and Video S1, Supporting Information) illustrate this scrolling process in real time.

To evaluate the influence of scrolling length on microscroll morphology, scrolls with lengths of 5, 10, 15, 20, and 25 mm were fabricated (Figure S6). As expected, longer scrolling length resulted in a greater number of lamellae and larger outer diameters. For instance, scrolls fabricated with 25 mm scrolling length exhibited up to 19 lamellae and a diameter of ∼700 µm (Figures 3d and S6), whereas scrolls of 5 and 10 mm displayed 6 and 10 lamellae, respectively. Cross‐sectional SEM analysis revealed well‐formed concentric architecture with Fe_3_O_4_ particles embedded between VNF layers, giving rise to slight localized surface waviness along the scroll profile (Figure 3d). The lamellae maintained structural continuity throughout, with no evidence of delamination or cracking. Higher magnification images revealed slight local kinking (micro‐buckling) in the innermost lamellae, consistent with the higher compressive stresses associated with small bending radii. Such localized deformations gradually diminish in outer lamellae as the curvature decreases. The observed deformation behavior is consistent with the bending‐induced stress distribution expected in a tightly wound multilamellar film: the innermost lamellae experience the highest curvature and therefore show the most pronounced local kinking, whereas the outer lamellae remain less deformed. The preservation of lamellar continuity and the absence of cracking or delamination indicate that the VNF network accommodates the imposed curvature while the embedded Fe_3_O_4_ particle‐rich domains remain mechanically integrated within the scroll architecture. This interpretation is consistent with the previously reported bending‐induced stress gradients and delamination–winding behavior of razor‐blade‐scrolled VNF‐based films [47].

Parallel fabrication of multiple microscrolls with well‐defined widths was achieved using razor blades modified into comb‐like geometries (Figure S7, Supporting Information). Blades with 2 or 5 mm width enabled simultaneous scrolling of up to eight (2 mm) or four (5 mm) individual microscrolls, with scrolling lengths ranging from 3 mm to 25 mm (Figures 3e and S9, Supporting Information). Scrolls with a width of 2 mm exhibited a faster and more reproducible magnetic response under identical NdFeB magnet conditions, likely due to more efficient coupling to the local magnetic field gradient. Therefore, 2 mm wide scrolls were selected for subsequent magneto‐mechanical experiments. Wider 5 mm scrolls displayed a weaker magnetic response under the present field configuration. The actuation threshold should therefore be regarded as an effective threshold for a given scroll geometry and magnetic‐field configuration, rather than as a geometry‐independent material constant. In the present setup, the reliable actuation distance is limited by the field strength and field gradient generated by the single permanent magnet. Accordingly, the working distance and actuation range could be further increased by using stronger magnets, optimized magnet geometries, or externally controlled magnetic‐field configurations.

The root region of the microscroll can act as a stress‐concentration site under uncontrolled deformation. Preliminary manual handling showed that off‐axis pulling or uncontrolled opening of as‐prepared microscrolls on the original substrate could lead to detachment at the root. However, no root fracture or detachment was observed during controlled opening using a micrometer stage or during magnetic actuation experiments performed on microscrolls fixed onto glass support substrates under defined conditions. Beyond individual scroll formation, the comb‐like blade approach provides a reproducible linear‐contact geometry that supports controlled microscroll fabrication across defined film areas. Because the process is based on repeated linear contact geometries rather than individual manual rolling, it is compatible with larger array‐based actuator architectures and could, in principle, be adapted to roll‐to‐roll processing.

### 2.4 Magnetic Field Threshold and Multidirectional Actuation of VNF/Fe_3_O_4_ Microscrolls

**FIGURE 4:**
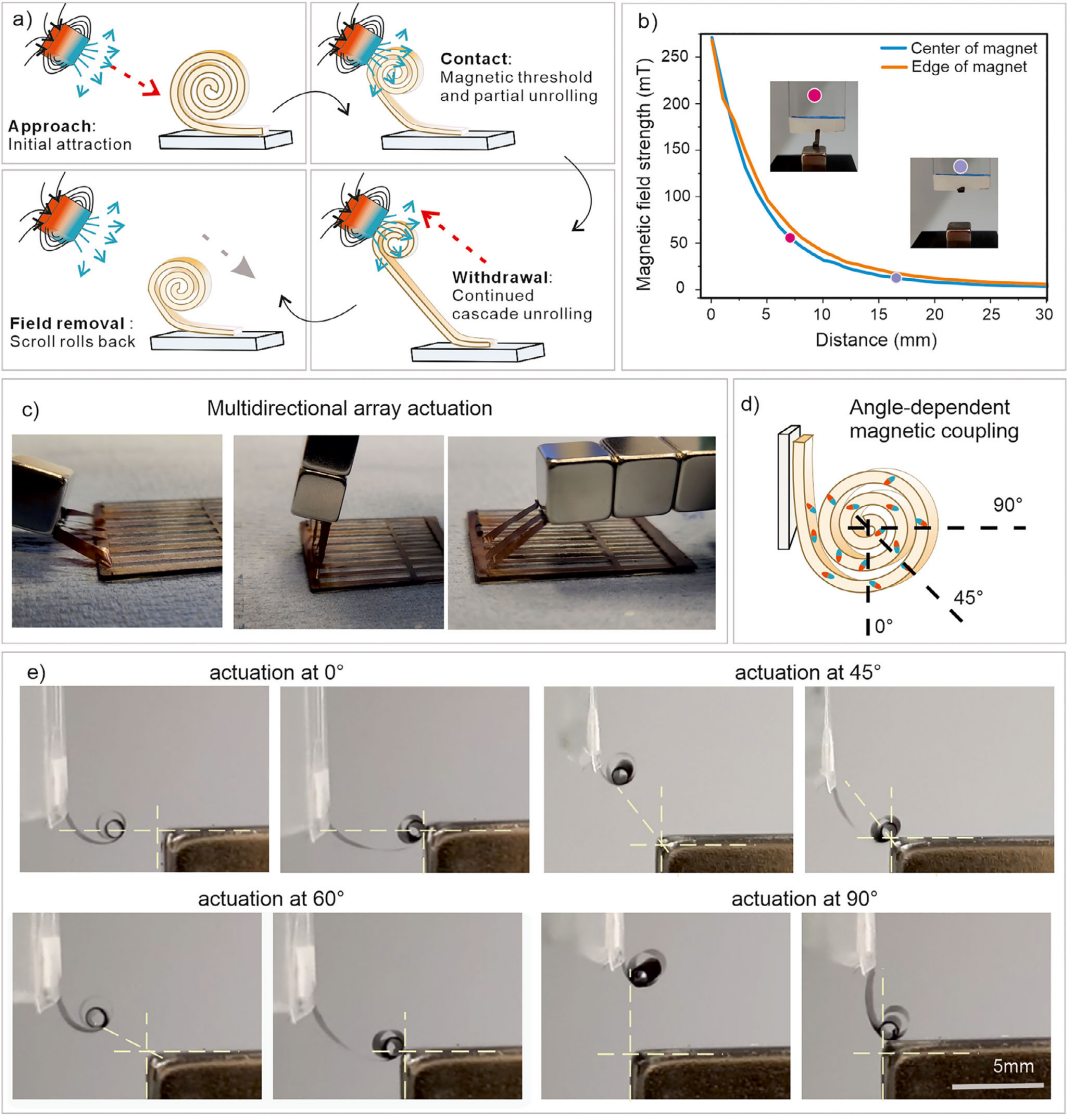
Magnetic field‐induced actuation and directional control of VNF/Fe_3_O_4_ microscrolls. (a) Schematic illustration of the actuation sequence under a moving permanent magnet. (b) Magnetic field strength as a function of distance with markers indicating the experimentally observed actuation threshold. (c) Multidirectional actuation of a microscroll under magnetic fields applied along three different directions within the plane perpendicular to the scroll axis (curvature plane), illustrating the continuous 180° azimuthal actuation range (Video S2, Supporting Information). (d) Schematic illustration of angle‐dependent magnetic coupling in the microscroll architecture. (e) Representative image sequences showing actuation of a single microscroll at opening angles of 0°, 45°, 60°, and 90°.

The actuation behavior of the microscroll follows a characteristic progression governed by its spatial relationship to the external magnetic field. These steps are illustrated schematically in Figure 4a.

As the magnet approaches, the scroll begins to unroll under the influence of magnetic attraction. Upon contact with the magnet, localized magnetic torque drives further unrolling. Continued unrolling occurs as the magnet is gradually retracted. Finally, when the magnetic field is removed, the scroll rapidly returns to its original coiled configuration. To determine the minimum magnetic field strength required to initiate scroll actuation, we first characterized the spatial magnetic field distribution of a NdFeB permanent magnet (10 mm × 10 mm × 10 mm) using a Hall‐probe Gaussmeter. Measurements performed at controlled distances from both the center and edge of the magnet, ranging from 0 mm to 30 mm, revealed predictable spatial variations in field strength (Figure 4b), with values decreasing significantly with distance and showing small but measurable differences between the magnet center and edge.

To assess the actuation threshold experimentally, a VNF/Fe_3_O_4_ microscroll (20 mm, 11 wt% Fe_3_O_4_) was fixed to a glass substrate and incrementally advanced toward the magnet using a micrometer‐controlled positioning setup. The scroll remained in its resting configuration until it approached within ∼7 mm of the magnet surface, at which point a clear actuation response was observed. Based on the magnetic field distribution shown in Figure 4b, this distance corresponds to a field strength of approximately 60 mT at the edge and 50 mT at the center of the magnet. We note that the exact actuation distance varies slightly between individual scrolls (typically by ± 0.5–1 mm), consistent with minor variations in local Fe_3_O_4_ loading and thus in the effective magnetization, as discussed in Section 2.2.

The interaction force between the magnet and the scroll can be described in terms of the field‐induced magnetization of the scroll and the magnetic‐field gradient. This macroscopic description includes the combined effect of distributed magnetic body forces arising from the Fe_3_O_4_ phase, including gradient‐driven attraction, magnetic‐moment alignment, and torque associated with the field‐induced magnetic moment, which cannot be individually resolved with the present characterization methods. Because the Fe_3_O_4_ nanoparticles in the hybrid film already reach a large fraction of their magnetic saturation at fields of ∼50–60 mT (Figure 2b), their magnetization varies less strongly than the field gradient near the actuation threshold. Consequently, the rapid rise in near‐field scroll–magnet attraction as the magnet approaches is primarily governed by the magnetic field gradient. Approximating the permanent magnet as a magnetic dipole, the magnetic field decreases approximately as B∝ d^−3,^ while the field gradient scales as ∇B ∝ d^−4^. In the linear magnetization regime of the nanoparticles (M ∝ H), the resulting scroll–magnet force becomes F∝ d^−7^. Near partial saturation, this distance dependence becomes less steep but remains strongly governed by the field gradient. This steep near‐field dependence explains why the scroll is highly responsive to nearby magnets yet remains insensitive to weak or spatially uniform background fields, including the Earth's magnetic field. We note that the present actuation geometry relies on both the gradient‐driven attraction and magnetic torque associated with the embedded Fe_3_O_4_ phase, therefore actuation under a spatially uniform field has not yet been demonstrated.

To enhance the effective magnetic field and enable simultaneous actuation of multiple scrolls, four permanent magnets were arranged in a row configuration. Under these conditions, scroll actuation occurred at distances exceeding 7 mm, indicating a stronger cumulative magnetic field (Figure S8). Multiple scrolls were actuated simultaneously, demonstrating the feasibility of array‐based magnetic control. The unrolling motion was further evaluated by changing the relative orientation between the magnet and the microscroll. The scroll could be actuated over a broad angular range within its curvature plane, demonstrating multidirectional magnetic responsiveness. Bending of the outer lamellae, which initiates unrolling, is mechanically favored within the curvature plane perpendicular to the scroll axis. Consequently, when the magnetic field acts within this plane, the outer lamellae can bend toward the field source over a continuous azimuthal range, giving rise to the 180° multidirectional actuation shown in Figure 4c and Video S2. In contrast, magnetic fields applied approximately parallel to the scroll axis predominantly promote whole‐scroll translation or alignment toward the magnet, whereas efficient lamellar opening requires field coupling within the curvature plane. The observed multidirectional actuation therefore arises from the combination of field‐responsive Fe_3_O_4_ nanoparticles and geometrically constrained deformation of the multilamellar scroll architecture. This multidirectional opening behavior differs from the predominantly uniaxial or bidirectional bending modes typically reported for thin‐film magnetic actuators [3, 4].

To further analyze the multidirectional actuation behavior, the response of a single microscroll was examined at opening angles of 0°, 45°, 60°, and 90° (Figure 4d). The magnet–scroll distance required to induce actuation increased from 3.9 mm at 0° to 4.7 mm at 45°, 5.1 mm at 60°, and 6.1 mm at 90°, corresponding to approximate magnetic field ranges of ∼105–115 mT, ∼85–95 mT, ∼75–85 mT, and ∼60–70 mT, respectively. Because these values were extracted from video frames using the magnet width as an internal scale, they should be interpreted as semi‐quantitative indicators of the angular dependence of the actuation threshold. The observed trend confirms that the 180° multidirectional actuation depends on the relative field–scroll orientation. Thus, the response is not isotropic in all spatial directions, but is governed by angle‐dependent magnetic coupling within the mechanically accessible curvature plane of the scroll. The actuation trajectory therefore results from the balance between magnetic torque, elastic restoring torque stored in the curved lamellae, and interlamellar resistance.

The comb‐like blade approach enables parallel fabrication of multiple microscrolls on a single substrate, with scroll lengths ranging from 3 mm to 25 mm (Figure S9). Microscrolls with the same scrolling length showed qualitatively similar actuation behavior under comparable magnetic‐field conditions. In the present setup, simultaneous actuation of all scrolls in one row was limited by the 10 × 10 × 10 mm permanent magnet, which did not provide identical field conditions across the full array of approximately 2 mm‐wide scrolls. Larger or more homogeneous magnetic‐field sources should therefore enable collective actuation of larger arrays (Figure S10). In addition, combining scrolls of different lengths within one substrate offers a route toward geometry‐programmed actuation patterns. Future scaling toward larger‐area or wafer‐scale fabrication will require improved control of film thickness, drying conditions, blade alignment, and field homogeneity.

### 2.5 Dual Mechanism of Magnetic and Mechanical Actuation in VNF/Fe_3_O_4_ Microscrolls

**FIGURE 5:**
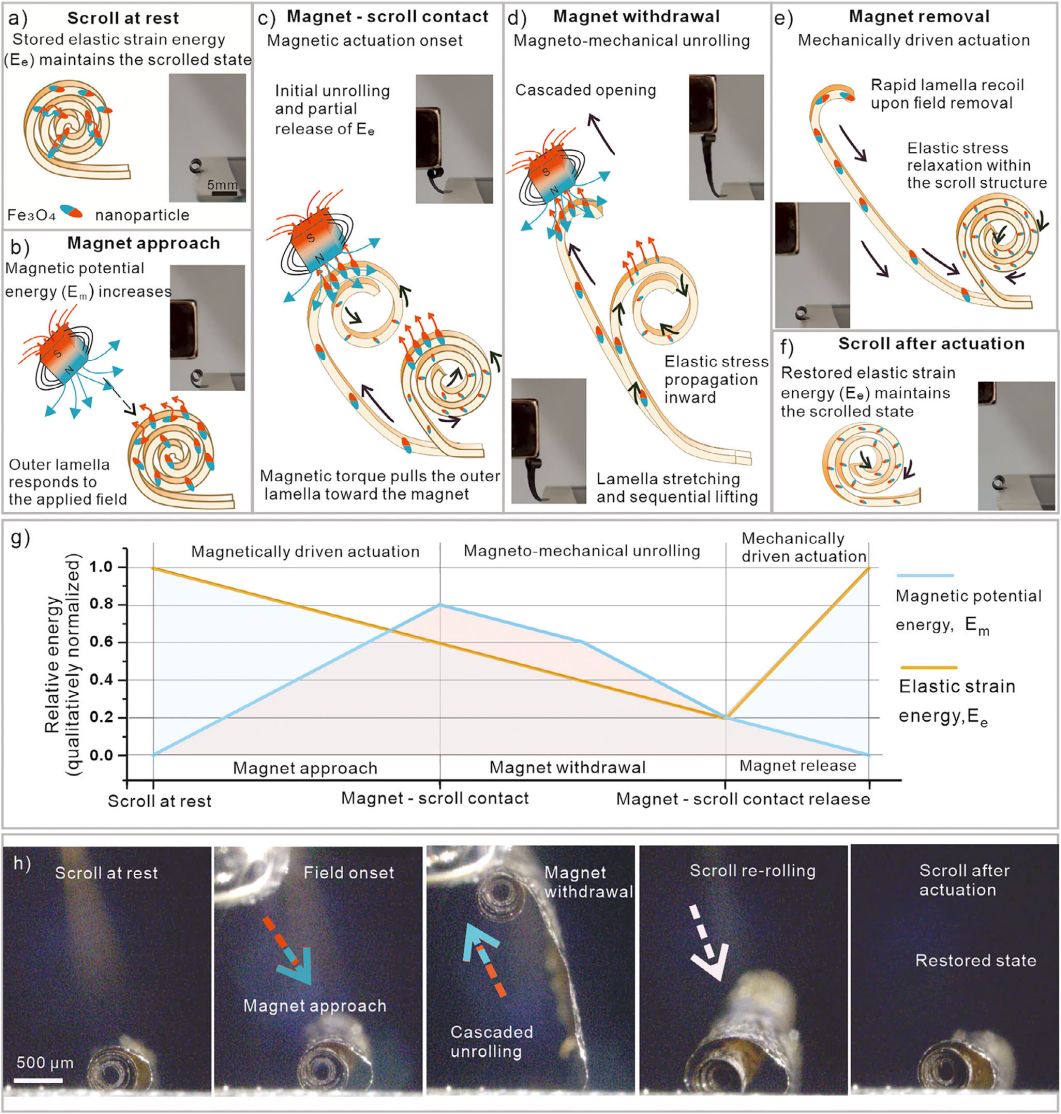
Dual magneto‐mechanical actuation mechanism in VNF/Fe_3_O_4_ microscrolls. (a–f) Schematic representations and digital side‐view images (10 mm scroll, Video S3, Supporting Information) illustrating the sequential stages of actuation: resting state, magnetic field onset, magnetic attraction, cascaded unrolling during magnet withdrawal, field removal, and recoil. (g) Normalized energy diagram showing the qualitative evolution of magnetic potential energy (*E*_m_) and elastic strain energy (*E*_e_) throughout the cycle, highlighting the cooperative dual‐energy behavior. The energy diagram is qualitative and illustrates the relative evolution of magnetic and elastic contributions rather than an absolute energy balance. (h) In situ microscopic observation of the actuation cycle in a VNF/Fe_3_O_4_ microscroll, showing sequential stages of actuation. Lamellae slide smoothly over the magnet surface, confirming non‐adhesive field‐mediated actuation (Video S4, Supporting Information).

The observed actuation behavior confirms that the Fe_3_O_4_ nanoparticles embedded within the VNF lamellar matrix support a coordinated magnetic response, resulting in reversible and mechanically stable motion. Importantly, while the magnetic response of the superparamagnetic filler is field‐induced and does not require programmed magnetic anisotropy, the mechanical deformation mode is dictated by the scroll geometry: unrolling proceeds primarily within the curvature plane. Beyond magnetic responsiveness, the scroll geometry itself plays an active role during actuation. The mechanical scrolling process stores residual elastic strain energy within the multilamellar film structure. Upon removal of the magnetic field, this stored energy drives rapid recoil, restoring the scroll to its coiled configuration. Based on real‐time optical tracking (Video S3, Supporting Information), the actuation cycle can be divided into four principal stages: (1) magnetic field onset, (2) magnet–scroll contact, (3) magnet withdrawal, and (4) field release. These stages are illustrated schematically in Figure 5a–f, with corresponding optical side‐view images of a 10 mm scroll, confirming each step.

In the absence of a magnetic field (resting state, Figure 5a), the scroll remains tightly coiled, consisting of multiple rolled lamellae formed during blade‐assisted fabrication. In this state, the scroll contains stored elastic strain energy (*E*_e_), while embedded Fe_3_O_4_ nanoparticles carry no net remanent magnetization and the magnetic contribution *E*_m_ is effectively zero.

The actuation begins with the field‐onset phase (Figure 5b). Upon application of an external magnetic field, Fe_3_O_4_ nanoparticles acquire magnetic moments, generating distributed magnetic stress superimposed on the pre‐existing elastic stress field. Initially, the entire scroll is attracted toward the magnet (Figure 5c), exhibiting a collective volumetric response due to its magnetic nanoparticle density and compact packing. Similar cooperative motion of magnetically responsive multilayered films has been reported by Xu et al. [49].

As *E*_m_ increases, the Fe_3_O_4_ phase develops a field‐induced magnetic moment, generating a magnetic torque that acts on the magnetically loaded lamellae. This effect is most relevant at the outer lamellae, where local bending and partial release of the stored elastic strain energy can initiate unrolling. During this transition, the elastic restoring force opposes the torque, defining the mechanical balance that governs the onset of actuation. During contact with the magnet surface, the magnetic contribution reaches its highest value (because of the smallest distance between lamella and magnet), while the elastic strain energy is only partially released because the inner lamellae retain substantial bending energy. Thus, the magnetic field acts primarily as a local trigger for outer‐lamella release rather than as the sole energy source for complete scroll unrolling.

As the magnet is withdrawn in the direction of unrolling (Figure 5d), the unrolling front propagates stepwise from the outer lamellae toward the inner lamellae. The outermost lamella responds first, followed sequentially by the inner lamellae as the region of the maximum field gradient shifts along the scroll. Each lamella unrolls when the local magnetic contribution becomes sufficient to overcome its elastic resistance, giving rise to a cascaded lamella‐by‐lamella unrolling process. Maintaining the magnet near the leading edge of the scroll preserves optimal field intensity and enables sustained directional actuation.

Once the magnetic field is removed, the residual *E*_e_, which is distributed across the scroll but largest in the inner lamellae, drives spontaneous re‐rolling of the scroll (Figure 5e) and the structure returns to its initial state without external input (Figure 5f). This sequence reflects a coupled magneto‐mechanical actuation pathway, in which distributed magnetic stresses from the Fe_3_O_4_ phase trigger motion, while stored elastic strain energy in the multilamellar scroll contributes to cascade propagation and rapid recoil. The normalized energy diagram in Figure 5g depicts the qualitative evolution of these energies throughout the cycle emphasizing relative trends rather than absolute magnitudes. This geometry‐assisted, energy‐coupled actuation pathway highlights a distinct actuation mode for ceramic‐based microscale magnetic actuators, in which magnetic triggering is coupled to the release of mechanically stored elastic energy.

To move beyond this qualitative description, we performed a quantitative comparison between the magnetic torque generated by the Fe_3_O_4_‐loaded scroll and the elastic restoring torque stored in the multilamellar geometry. While the mechanism is schematically represented in Figure 5g in terms of magnetic triggering and elastic energy release, the quantitative comparison was performed at the torque level because scroll opening proceeds through the unrolling of curved lamellae. The detailed quantitative evaluation is provided in Sections S14–S16 of the Supporting Information. Briefly, the analysis combines three experimentally parameterized inputs: SQUID magnetometry on individual 15 mm microscrolls to determine the absolute magnetic moment (Figure S11), Hall‐probe measurements of the magnetic field as a function of distance from the magnet (Figures 4b and S12a), and mechanical opening tests combined with an analytical bending model to estimate the elastic restoring contribution of the lamellae (Figure S13 and Tables S2 and S3).

The onset of magnetic unrolling was not assigned to a single universal threshold distance, because the apparent actuation threshold depends on the initial scroll geometry, packing density, and degree of stored elastic strain. In the actuation experiments, unrolling was commonly initiated when the magnet was positioned approximately 5–7 mm from the scroll edge. This distance range was therefore treated as the experimentally relevant onset window. In this analysis, the magnetic contribution is treated as an experimentally parameterized effective torque estimate rather than as a full coupled magneto‐mechanical simulation. The estimate combines the SQUID‐derived field‐dependent magnetic moment of an individual scroll with the measured Hall‐probe magnetic‐field profile to evaluate whether the available magnetic torque is sufficient to initiate release of the outer lamellae. It is therefore used as an order‐of‐magnitude comparison with the elastic restoring torque, while the relative roles of gradient‐driven attraction, distributed magnetic stresses, and contact friction remain unresolved. Using the measured magnetic field profile and the SQUID‐derived field‐dependent magnetic moment, the calculated magnetic torque in this onset window is on the order of 10^2^ nN m for the 15 mm scroll (Figure S12b and Table S1). At direct magnet contact, the calculated magnetic torque reaches approximately 576 nN m, defining the upper bound of the magnetic torque available during actuation.

To determine whether this magnetic torque is sufficient to overcome the elastic resistance of the scroll, we independently estimated the elastic restoring contribution from mechanical opening experiments on a 15 mm scroll combined with an analytical bending model (Figure S13). During mechanical unrolling, the measured force increased continuously to a peak load of approximately 3.5 mN before slip occurred between the scroll and the carbon rod. The analytical model, based on the measured cross‐sectional scroll geometry, predicts a cumulative restoring force of approximately 6.0 mN for complete engagement of all modeled lamellae, with a corresponding stored bending energy of approximately 13.7 µJ (Table S2). Converting the lamella‐resolved restoring forces into torques gives an elastic restoring torque of approximately *τ*_e, L1_ ≈ 215 nN m for the outermost lamella and a cumulative elastic torque of *τ*_e, total_ ≈ 2180 nN m for the full multilamellar structure (Table S3). Thus, the magnetic torque in the onset window is comparable to the restoring torque of the outermost lamella, but remains far below the cumulative elastic torque of the complete scroll (Figure S14). This comparison indicates that the magnetic field is sufficient to initiate local bending and partial release of the outer lamella, but cannot quasi‐statically overcome the full elastic resistance of the scroll. Complete and rapid unrolling therefore requires release of the stored elastic bending energy encoded in the multilamellar architecture. This upper‐bound torque exceeds the elastic torque of the outermost lamella and approaches the cumulative contribution of the first several outer layers, but remains well below the cumulative elastic torque of the tightly curved inner region (Figure S14). Therefore, the magnetic field initiates outer layer opening and guides the unrolling direction, whereas complete propagation of the unrolling front requires release of the stored elastic energy. This torque comparison explains why the scroll does not behave as a purely magnetically pulled object, but rather as a geometry‐programmed actuator in which the magnetic field acts as a trigger and directional guide.

High‐magnification in situ optical microscopy further supports this interpretation of contact‐assisted, torque‐guided unrolling. Although the scroll appears macroscopically to remain in direct contact with the magnet during actuation, optical observations reveal that it glides over the magnet surface rather than adhering to it. This behavior indicates that the contact stage is not governed by simple magnetic sticking, but by a dynamic balance between magnetic attraction, magnetic torque, elastic compliance, and interlamellar sliding. The image sequence in Figure 5h and Video S4 shows the scroll at rest, field onset, magnet–scroll contact, cascaded unrolling, spontaneous re‐rolling, and recovery of the coiled configuration. During this process, the lamellae slide smoothly over the magnet surface, confirming non‐adhesive, field‐mediated actuation. This observation is consistent with the torque analysis: at direct magnet contact, the magnetic torque is sufficiently high to initiate opening of the outer lamellae and guide the unrolling direction, while the subsequent cascade and recoil remain governed by the release of stored elastic bending energy. Such smooth sliding is consistent with energy‐minimizing dipolar interactions reported for magnetically responsive soft structures [50], but in the present scrolls it is additionally constrained by the multilamellar elastic geometry.

The same mechanism also explains the strong geometry dependence of actuation. For scrolls fabricated under the scrolling parameters used in this study, intermediate rolling lengths, in the range of 10–20 mm, exhibited the most efficient unrolling because their curvature, lamellar packing, and magnetic loading provide a favorable balance between magnetic torque and elastic resistance. According to beam bending theory [48], shorter and more tightly packed scrolls often required closer magnet proximity for the first opening event, consistent with their smaller effective radii and higher bending resistance, while their lower magnetic material content also reduces the available magnetic moment. After initial opening, subsequent actuation often occurred more readily, suggesting partial relaxation of stored elastic strain and an increase in the effective lamellar radius. In contrast, longer scrolls can unroll only partially when the outer lamellae move into regions of weaker magnetic field, where the available torque is no longer sufficient to sustain further propagation. The actuation window is therefore not defined by scroll length alone, but by the coupled effects of rolling length, internal radius, lamellar packing, magnetic moment, and residual elastic strain. This geometry dependence also provides a design opportunity: by adjusting scrolling parameters, the radius and packing density of the scroll can be tuned to program the actuation threshold and unrolling behavior. Consistent with this torque‐based interpretation, the apparent actuation threshold also depends on the relative orientation between the scroll and the magnetic field. In the multidirectional actuation experiments (Figure 4c), the magnet–scroll distance required to induce opening varied with the opening angle from 0° to 90°, confirming that the response is governed by angle‐dependent magnetic coupling within the mechanically accessible curvature plane of the scroll rather than by an isotropic magnetic attraction alone.

The combined SQUID‐derived torque estimation, mechanical opening experiment, analytical bending model, and geometry‐dependent actuation behavior support the proposed dual magneto‐mechanical mechanism. The field‐induced magnetic torque generated by the embedded Fe_3_O_4_ nanoparticles initiates release of the outer lamellae and guides the direction of unrolling, whereas the mechanically stored elastic strain energy in the multilamellar scroll provides the main energy reservoir for cascade propagation and rapid recoil. Unlike conventional soft magnetic actuators, where deformation is typically governed by a compliant polymer matrix or programmed magnetic anisotropy, this system stores and releases mechanical energy through a structurally encoded multilamellar ceramic‐based architecture. This geometry‐assisted coupling offers a route to rapid, reversible, and tunable actuation in mechanically robust ceramic thin‐film systems.

### 2.6 Performance Characteristics of the VNF/Fe_3_O_4_ Microscroll Actuator

The VNF/Fe_3_O_4_ microscroll actuators, fabricated with loadings of 8.6 and 11 wt% demonstrate a compelling combination of geometric compactness, mechanical compliance, and rapid magnetic responsiveness. Unless otherwise indicated, performance quantification focuses on the 11 wt% composition, which exhibited the most favorable balance between flexibility and magnetic response. All experiments were performed under ambient laboratory conditions of *T* = 22 ± 2°C and RH = 48 ± 5%. Because V_2_O_5_ nanofibers contain interlayer and surface‐bound water, strongly dry or high‐humidity conditions may alter the hydrogen‐bonded VNF lamellar network and thereby affect residual stress, curvature radius, and actuation performance. The values reported here therefore correspond to the stated ambient conditions.

A particularly notable geometric feature of the microscroll actuator is the remarkable footprint reduction achieved upon scrolling. A planar hybrid film (15 mm × 2 mm × ∼4.2 µm) forms a scroll with a diameter of ∼0.7 mm, corresponding to a > 90% decrease in projected area. Longer scrolls (20–25 mm) achieve even higher reductions, up to ∼96%, offering a clear spatial advantage for compact or microscale actuation. When evaluated geometrically, this transformation corresponds to a geometric expansion ratio of approximately 1400%, defined as the ratio between the unrolled scroll length and its rolled diameter. This metric reflects the extent of deployable motion enabled by the architecture and is distinct from engineering strain, which quantifies material‐level deformation. The microscroll therefore enables large‐amplitude geometric reconfiguration with minimal intrinsic material strain, an advantage for long‐term cycling durability. Each cycle involved full unrolling (120 ms), followed by rapid elastic recoil (30 ms), yielding a total actuation time of ∼150 ms (Video S6, Supporting Information). The slower unrolling phase reflects the balance between magnetic torque and the residual elastic stress that preserves the coiled geometry, as described in Section 2.5. Upon removal of the magnetic field, the release of this stored stress drives the rapid elastic recoil, enabling spontaneous and repeatable retraction. These dynamics are comparable to or faster than reported film‐based magnetic or multimodal actuators [22], and significantly exceed the response rates of thermally, pneumatically, or hydrogel‐driven systems [4]. Only around 10% of reported soft magnetic actuators demonstrate stable operation beyond 1000 cycles [3]. In contrast, our microscroll retained its lamellar integrity and reversible actuation capability throughout testing.

**FIGURE 6:**
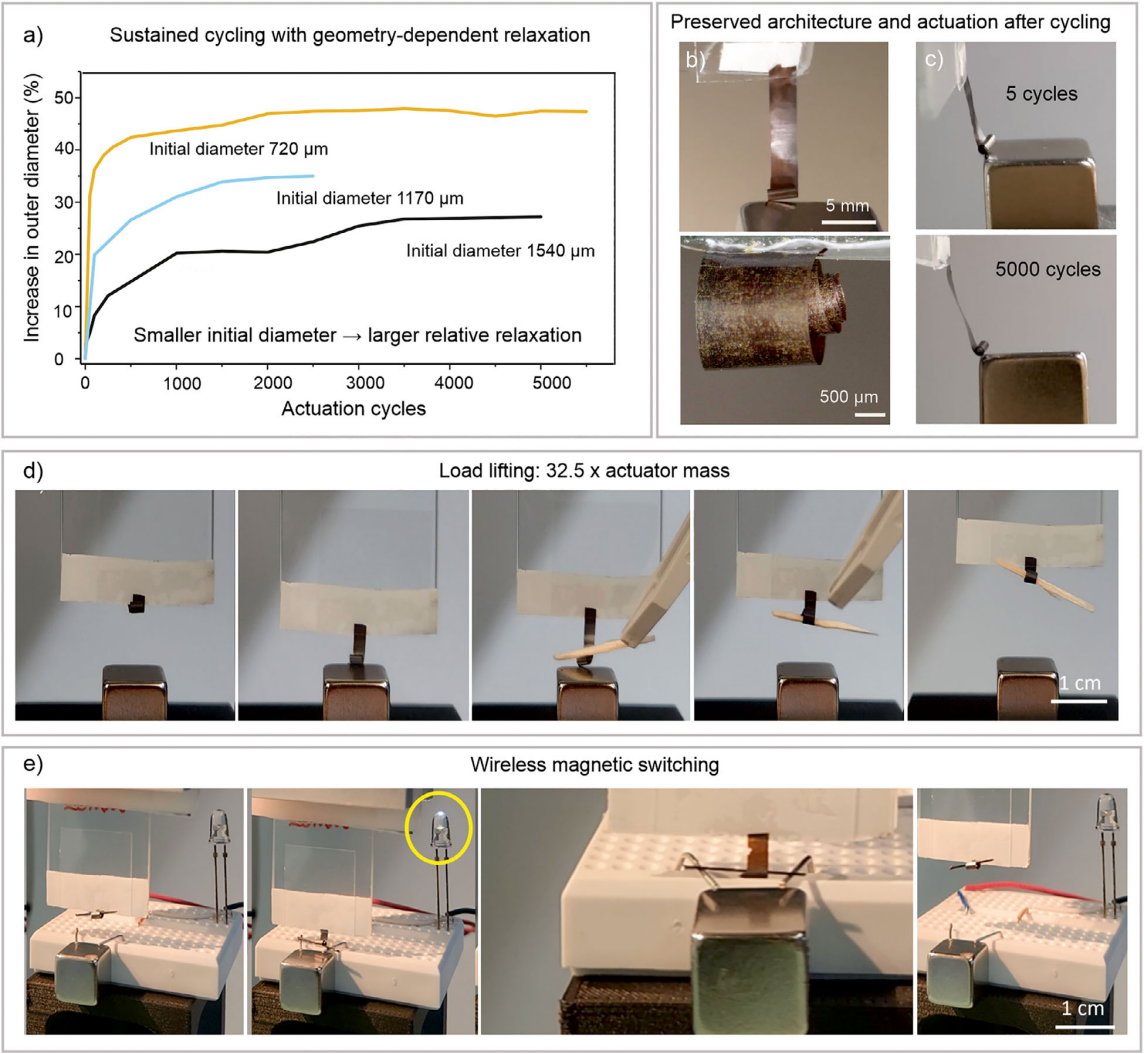
Cycling durability and functional performance of VNF/Fe_3_O_4_ microscroll actuators. (a) Relative increase in outer diameter as a function of magnetic actuation cycles for 20 mm‐long microscrolls. (b) Front and side views of the microscroll after 5000 actuation cycles, showing preservation of the inner core and outer scroll architecture. (c) Comparison of the opened scroll length after 5 and 5000 actuation cycles, showing retained magnetic responsiveness. (d) Load‐lifting demonstration using a 15 mm microscroll to lift a 13 mg wooden rod (Video S7, Supporting Information). (e) Wireless magnetic switching demonstration. A 20 mm scroll lowers a 4 mg carbon rod to close an electrical circuit and illuminate an LED (yellow circle). The inset shows the contact interface between the carbon rod and the metal pad. Upon magnet removal, the scroll recoils and breaks the circuit, enabling repeatable on/off switching (Video S8, Supporting Information).

To quantitatively assess the cyclic durability of the VNF/Fe_3_O_4_ microscroll actuators under repeated magnetic operation, we performed extended durability tests on 20 mm‐long microscrolls representing different degrees of initial compactness. Two representative scrolls with initial outer diameters, approximately 720 and 1540 µm were cycled for more than 5000 magnetic actuation cycles. The relative increase in outer diameter was tracked as a function of cycle number to evaluate geometry‐dependent relaxation during repeated unrolling and re‐rolling. Cross‐sectional optical microscopy images were recorded after selected cycle numbers, and the inner and outer scroll diameters were extracted to assess possible geometry changes during cycling (Figure S15, Supporting Information). As shown in Figure 6a, the dominant change occurs in the outer scroll diameter, which increases mainly during the initial cycling stage and then approaches a quasi‐stable state.

After 5000 cycles, the outer diameter increased by approximately 27% for the initially larger scroll and by approximately 47% for the more compact scroll. An additional microscroll with an intermediate initial outer diameter of ∼1170 µm was followed up to 2500 cycles and showed an intermediate diameter increase of ∼35%, supporting the same trend. These results indicate that cyclic actuation induces stronger lamellar relaxation in initially more tightly wound scrolls. In contrast, the inner scroll diameter remains largely preserved throughout the test, indicating that cyclic actuation primarily relaxes the outer lamellae rather than disrupting the compact inner core of the scroll.

Representative optical microscopy images (Figure S15, Supporting Information) confirm that the microscrolls retain their coiled multilamellar architecture after 5000 cycles, without visible fracture, delamination, or collapse of the scroll structure. One example is presented in Figure 6b. This behavior suggests that the observed increase in outer diameter reflects partial relaxation of the pre‐stored elastic strain and local accommodation of the outer lamellae, rather than fatigue‐induced failure. Consistent with this interpretation, image‐based comparison of the opened scroll configuration before and after extended cycling shows that the scroll remains magnetically responsive and can still undergo pronounced opening after 5000 cycles (Figure 6c). The slightly increased opened length after cycling is consistent with the measured increase in outer diameter and indicates that cyclic relaxation does not lead to attenuation of actuation capability within the tested range. Instead, repeated actuation results in limited geometric accommodation while preserving the functional scroll architecture.

The load‐bearing capability of the microscroll was evaluated using a 15 mm × 2 mm actuator with a mass of ∼0.4 mg. The scroll lifted a 13 mg wooden rod, corresponding to a lifting ratio of ∼32.5× the actuator mass (Figure 6d and Video S7, Supporting Information), and maintained stable grip even after being moved outside the magnetic field. The actuation distance (∼7 mm from the magnet surface) corresponds to a local magnetic field of ∼50–60 mT, consistent with the field‐dependent torque–curvature balance discussed in Section 2.5. Such lifting ratios exceed those commonly reported for untethered soft magnetic actuators, which typically remain below 10× and often struggle to maintain repeatable gripping performance across cycles [3, 51]. Although advanced composite systems can achieve extreme lifting ratios (∼1000×) using shape‐memory polymers and laser heating [13], those architectures require complex dual‐stimulus strategies and are not directly comparable to passive, field‐driven microscale actuators such as ours.

Based on the measured displacement (Δ*y* ≈ 8 mm) and lifted mass (13 mg), the microscroll performs 1.02 µJ of mechanical work per cycle. Using the unrolled film volume (1.26 × 10^−^^10^ m^3^) as the normalization basis, which provides a consistent reference geometry for comparing film‐based actuators, this corresponds to a work density of ∼8.1 kJ m^−^^3^. If the rolled volume were used instead, the value would be substantially higher. However, this geometry is not representative for benchmarking and would artificially inflate performance metrics. We therefore report only the unrolled‐volume‐based value to maintain consistency with established conventions [1, 51]. The magnetic‐to‐mechanical energy exchange during actuation was further evaluated using an apparent magneto‐mechanical amplification factor, *Λ*, defined as the ratio of the mechanical output work during one actuation stroke to the magnetic‐field‐associated trigger energy estimated for the actuator volume. Importantly, *Λ* should not be interpreted as a classical thermodynamic efficiency or as a direct magnetic‐to‐mechanical energy‐conversion ratio. In the present microscroll, a substantial fraction of the mechanical work originates from elastic strain energy pre‐stored during the mechanical scrolling/fabrication step. The magnetic field applied during operation acts primarily as a trigger or modulator that releases this pre‐stored elastic energy through alignment of the field‐induced magnetization of the embedded Fe_3_O_4_ particles. Consequently, *Λ* may exceed unity without violating energy conservation, because the one‐time mechanical energy input required to form the pre‐stressed scroll is not included in the operational magnetic trigger energy. The obtained value of *Λ* ≈ 5.6 therefore reflects the leverage of the magnetic trigger over a mechanically pre‐stressed architecture, rather than an energy‐conversion efficiency. A complete quantitative energy balance including the scrolling/fabrication work, stored elastic strain energy, magnetic trigger energy, and cycle‐dependent dissipation would require dedicated mechanical modeling and is beyond the scope of this study.

At the system level, the actuator delivered a power output of 6.8 µW at a cycling frequency of 6.67 Hz, corresponding to a specific power of ∼17 W kg^−^^1^. The specific work (2.6 J kg^−^^1^) and actuation stress (∼15.2 kPa) fall within or exceed performance reported for other thin‐film magnetic actuators at comparable scales [2, 10, 52]. The combination of high work density, rapid elastic recoil, and stable µW‐level power output positions the microscroll as a promising platform for micro‐robotic systems requiring fast, repeatable, and untethered mechanical actuation.

To demonstrate functional versatility, the microscroll actuators were integrated into two proof‐of‐concept systems. In the load‐lifting experiment (Figure 6d), the scroll mechanically engaged and lifted a wooden rod, while in the magnetic switch prototype (Figure 6e), a 20 mm scroll lowered and raised a 4 mg carbon rod to reversibly open and close an electrical circuit. In both cases, the microscroll was handled, positioned, and mechanically coupled to a passive component using simple assembly steps, illustrating compatibility with conventional pick‐and‐place workflows for soft‐robotic and microsystem applications. An additional gripping demonstration using strip‐type actuators fabricated from the same VNF/Fe_3_O_4_ hybrid film is provided in Figure S16, highlighting the broader design space of the material platform beyond the microscroll architecture.

Overall, the performance characteristics of the VNF/Fe_3_O_4_ microscrolls arise from the synergy between ceramic‐based mechanical robustness, nanoscale magnetic responsiveness, and geometry‐programmed elastic energy storage. Unlike conventional soft‐matter‐based magnetic actuators, the present system combines high load‐bearing capability at the microscale, rapid elastic recoil without continuous external input, high work density, stable cyclic performance over 5000 actuation cycles, and compact deployable configurations with up to 96% footprint reduction. Beyond the proof‐of‐concept demonstrations presented here, the combination of compact multilamellar geometry, rapid untethered magnetic responsiveness, reversible opening behavior, and mechanical durability may enable future applications in microscale gripping systems, remotely triggered switches, adaptive soft robotic components, and array‐based reconfigurable actuator architectures. These capabilities are particularly relevant to lab‐on‐a‐chip and microrobotic systems, where untethered microscale actuators capable of localized object manipulation and pick‐and‐place‐type operations are of considerable interest [10, 23].

To further contextualize the present microscrolls within the field of magnetic and magnetically responsive soft actuators, comparative benchmarking with representative systems is provided in Tables S4 and S5 (Supporting Information). The comparison highlights the distinctive combination of multilamellar ceramic architecture, rapid untethered magnetic actuation, multidirectional opening, mechanical robustness, and load‐bearing capability enabled by the present geometry‐programmed actuator design. More complex application scenarios, such as confined‐space operation, liquid‐environment actuation, micro‐particle transport, sequential crawling, or closed‐loop control, will require dedicated device‐level optimization and are identified as future opportunities.

## 3 Conclusion

Inspired by the compact, coiled geometry of the butterfly proboscis, we have developed a robust, scalable platform for magnetic soft actuators based on flexible V_2_O_5_/Fe_3_O_4_ hybrid films structured into tightly wound microscrolls. These actuators combine microscale flexibility enabled by the nanoscale fibrous network, strong magnetic responsiveness, and mechanical durability supported by the robust layered ceramic matrix, formed via a facile self‐assembly route. Fe_3_O_4_ nanoparticles are incorporated within the aligned V_2_O_5_ nanofibers without compromising film stiffness (∼30 GPa), while contributing to enhancing toughness through energy‐dissipating mechanisms at particle‐rich domains and nanoparticle–matrix interfaces.

Using a razor blade‐assisted scrolling method, the hybrid films are transformed into cylindrical microscrolls with tunable geometry and a multilamellar architecture. These actuators exhibit rapid and reversible motion under a near‐field permanent magnet stimulation (∼60 mT, ∼7 mm), including multidirectional bending, controlled unrolling and spontaneous recoil. Detailed investigation reveals a dual magneto‐mechanical actuation mechanism in which distributed magnetic stresses generated by the Fe_3_O_4_ phase trigger and drive unrolling, while stored elastic strain energy, introduced during scrolling, drives fast recoil and recovery of the coiled configuration. This cooperative interaction between scroll geometry, magnetic triggering, and stored elastic energy provides an effective route for amplifying actuation in compact magnetic microsystems.

Performance evaluation under proof‐of‐concept conditions demonstrated up to 96% footprint reduction, a geometric expansion ratio of ∼1400%, fast actuation (< 150 ms), and long‐term durability over 5000 cycles without loss of reversible actuation capability. A lifting ratio of ∼ 32.5× the actuator mass and an estimated work density of ∼8.1 kJ m^−^^3^ highlight the potential of these microscrolls as high‐performance ceramic based magnetic actuators at the microscale. Functional demonstrations illustrate their utility in untethered object manipulation, magnetic switching, and adaptive gripping.

Mechanical opening experiments combined with an analytical bending model further confirmed that the scroll stores a mechanically significant amount of elastic strain energy during formation. The SQUID‐derived magnetic torque is sufficient to initiate local bending and partial release of the outer lamellae, but remains below the cumulative elastic torque of the full multilamellar structure. This quantitative torque comparison supports the proposed dual mechanism, in which magnetic input acts as a trigger and directional guide, while stored elastic strain energy drives cascade‐like unrolling and rapid recoil.

Taken together, this work establishes a ceramic‐based microscroll actuator architecture and demonstrates how geometry‐programmed elastic energy can be coupled with magnetic torque to enhance actuation performance. The concept is broadly applicable to other nanostructured films and offers a pathway toward energy‐efficient microactuators for soft robotics, micromechanical systems, and a range of future application‐specific device platforms. A key next step toward real‐world applicability is to demonstrate actuation under spatially uniform or remotely controllable electromagnetic fields, such as Helmholtz‐coil or rotating‐field configurations. In addition, time‐resolved magnetometry under cyclic fields, magneto‐optical imaging, or synchrotron‐based probes such as time‐resolved XMCD or Fe L‐edge x‐ray microscopy could help distinguish magnetic‐moment alignment, local particle‐level torque, distributed magnetic stresses, and gradient‐driven body‐force contributions. These studies will be important for translating the present materials‐and‐mechanism concept into more advanced controlled microsystems.

## 4 Experimental Section

### 4.1 Synthesis of V_2_O_5_ Nanofibers

V_2_O_5_ nanofiber (VNF) stock solution was synthesized following a previously reported ion‐exchange method [36]. In brief, 1 g of ammonium metavanadate (NH_4_VO_3_, Fluka) and 10 g of hydrogen‐form ion exchanger resin (AMBERCHROM 50WX8, 50–100 mesh, Thermo Fisher Scientific) were added to 200 mL of deionized water and stirred in an oil bath at 80°C for 10 min. The resulting yellow solution was cooled to room temperature and stored in the dark. After 28 days, V_2_O_5_ nanofibers that had gradually formed through polycondensation were collected by decantation to separate them from the ion‐exchange resin. The concentration of the resulting suspension was 3.55 mg mL^−^^1^.

### 4.2 Synthesis of Fe_3_O_4_ Nanoparticles

Fe_3_O_4_ nanoparticles were synthesized via a solvothermal method. First, 0.97 g of FeCl_3_·6H_2_O was dissolved in 20 mL of ethylene glycol under stirring at room temperature for 2 h. Then, 0.01 g of trisodium citrate and 2.46 g of sodium acetate were added, and the mixture was stirred for additional 2 h. The solution was transferred to a Teflon‐lined stainless‐steel autoclave and heated to 200 °C at a rate of 1 °C min^−^^1^, followed by a 6 h reaction time. After cooling to room temperature, the black Fe_3_O_4_ precipitate was separated by centrifugation (4000 rpm, 10 min), washed six times with ethanol, and dried under vacuum at 55°C for 24 h. Aqueous suspensions (1–5 mg mL^−^^1^) of Fe_3_O_4_ nanoparticles were prepared, and TEAOH (Sigma‐Aldrich) was added as a surfactant in a 3:1 mass ratio (Fe_3_O_4_:TEAOH). The suspension was ultrasonicated for 30 min.

### 4.3 Fabrication of VNF/Fe_3_O_4_ Hybrid Films

To prepare the hybrid films, 1 mL of Fe_3_O_4_ aqueous dispersion was mixed with 9 mL of vanadium pentoxide nanofiber (VNF) dispersion and ultrasonicated for 30 min to ensure homogeneous mixing. Two different Fe_3_O_4_ concentrations were used depending on the targeted final composition: 3 mg mL^−^^1^ for films containing 8.6 wt% Fe_3_O_4_, and 4 mg mL^−^^1^ for films with 11 wt% Fe_3_O_4_. The resulting 10 mL hybrid dispersion was drop‐cast onto a rectangular glass substrate (20 mm × 60 mm) positioned centrally in a 50 mm‐diameter glass beaker. Slow, ambient drying (*T* = 22 ± 2°C and RH = 48 ± 5%) allowed water evaporation to drive self‐assembly of the nanofibers and nanoparticles into a layered hybrid architecture. After complete drying, a flexible and free‐standing VNF/Fe_3_O_4_ hybrid film was obtained.

### 4.4 Fabrication of Microscrolls

Microscrolls were fabricated using a razor blade‐assisted scrolling method previously established in our lab. Full technical details of the scrolling setup and fabrication process are available in our previous publication [47]. A razor blade was fixed at a 30° angle and gently brought into contact with the hybrid film, which was mounted on a linear translation stage moving at 1 mm s^−^^1^. A weight of 147 g was applied on top of the blade to ensure consistent pressure. Upon contact, the blade induced controlled delamination of the film, which rolled into a scroll as the substrate advanced along the stage. To enable parallel fabrication and controlled scroll width, custom laser‐cut comb‐like blades with 2 and 5 mm segments were used. Scrolling lengths between 3 mm and 25 mm were investigated.

### 4.5 Cyclic Durability Testing

The microscroll was mounted on a glass slide as described for the magnetic actuation experiments. A motorized translation stage was used to move the slide vertically at controlled speeds relative to the permanent magnet. Each cycle consisted of a controlled approach of the scroll toward the magnet at 0.5 cm s^−1^ until the actuation distance was reached, followed by rapid scroll unrolling. The sample was then retracted from the magnet at 0.2 cm s^−1^ until elastic re‐rolling occurred. The maximum number of actuation cycles was 5500. Optical microscopy images were recorded after 50, 100, and 250 cycles, and subsequently at 500‐cycle intervals from 500 to 5500 cycles, to determine the inner and outer diameters of the scrolls.

### 4.6 Object Lifting Test

A microscroll actuator (11 wt% Fe_3_O_4_ hybrid film, thickness 4.2 µm; width 2 mm; scrolling length 20 mm; mass 0.4 mg) was mounted on a glass slide using double‐sided adhesive tape and partially covered with a coverslip to provide mechanical support. A neodymium magnet (NdFeB, 10 mm × 10 mm × 10 mm, W‐10‐N, Supermagnete) was positioned below the scroll. A motorized translation stage was used to move the slide vertically at controlled speeds (0.5 cm s^−1^ downward, 0.2 cm s^−1^ upward). Once the scroll fully unrolled in response to the magnetic field, a 13 mg wooden rod was inserted into the innermost winding.

### 4.7 Light‐Switching Demonstration

A 15 mm‐long microscroll actuator (2 mm width, ∼4 µm thick, 11 wt%Fe_3_O_4_) was used to demonstrate wireless magnetic switching. A 4 mg carbon rod (0.5 mm diameter, 11 mm length) was inserted into the scroll. When exposed to the magnetic field generated by a 10 mm × 10 mm × 10 mm NdFeB magnet placed underneath, the scroll extended and lowered the rod to close an electric circuit and illuminate an LED. Upon removal of the field, the scroll recoiled and lifted the rod, opening the circuit. The switching process was fully reversible and repeatable.

### 4.8 Microstructural Characterization

The scrolling process was recorded using a digital light microscope (Keyence VHX‐2000). Cross‐sectional imaging of the microscrolls was performed using a Zeiss GeminiSEM 500 operated at 3 kV. For elemental analysis, energy‐dispersive x‐ray spectroscopy (EDX) was conducted using an XFlash Detector 630 M (Bruker Nano GmbH) at accelerating voltages ranging from 4 kV to 15 kV, depending on the elements being analyzed. The samples were sputter‐coated with a 2.5 nm iridium layer using an EM ACE600 coater (Leica Microsystems).

### 4.9 Mechanical Properties Characterization

Tensile properties of the VNF/Fe_3_O_4_ hybrid films, including Young's modulus, tensile strength, toughness, and fracture strain, were measured using a BOSE ElectroForce 3200 Series III mechanical tester. Hybrid films were cut into strips of 15 mm × 1 mm and tested at a strain rate of 0.001 mm s^−^^1^ with a load resolution of 0.15 mN. A total of 15 samples per composition were tested to ensure statistical reliability. The preparation method for tensile test specimens followed the procedure described in our previous publication [43]. Film thickness was determined by cross‐sectional SEM imaging after fracture. To quantify the mechanical force required to initiate scroll opening, tensile‐unwinding experiments were performed on individual microscrolls using a Nano Bionix mechanical testing system (Keysight Technologies; load resolution 50 nN). A thin carbon rod was gently inserted into the innermost winding of a 15 mm‐long scroll and pulled axially at a constant strain rate of 1 × 10^−^^4^ mm s^−^^1^. The force–displacement response was recorded throughout the test, enabling estimation of the lamellar uncoiling force and the corresponding internal mechanical stress stored within the scroll architecture. Unless otherwise stated, all mechanical and actuation experiments were performed under the same ambient laboratory conditions of *T* = 22 ± 2°C and RH = 48 ± 5%.

### 4.10 Magnetic Properties Characterization

Magnetic measurements were performed on a Quantum Design MPMS3 SQUID magnetometer. The magnetic field distribution of the NdFeB magnets used in actuation experiments was mapped using a Lakeshore 425 Gaussmeter by positioning a Hall probe at defined distances from both the center and the edge of the magnet.

## Funding

Deutsche Forschungsgemeinschaft (DFG) project number BU 2713/2‐3.

## Conflicts of Interest

The authors declare no conflicts of interest.

## Supporting information




**Supporting File 1**: adma74544‐sup‐0001‐Suppmat.pdf.

**Supporting File 2**: adma74544‐sup‐0002‐VideoS1.avi.

**Supporting File 3**: adma74544‐sup‐0003‐VideoS2.mp4.

**Supporting File 4**: adma74544‐sup‐0004‐VideoS3.mp4.

**Supporting File 5**: adma74544‐sup‐0005‐VideoS4.mp4.

**Supporting File 6**: adma74544‐sup‐0006‐VideoS5.mp4.

**Supporting File 7**: adma74544‐sup‐0007‐VideoS6.mp4.

**Supporting File 8**: adma74544‐sup‐0008‐VideoS7.mp4.

**Supporting File 9**: adma74544‐sup‐0009‐VideoS8.mp4.

## Data Availability

The data that support the findings of this study are available on request from the corresponding author. The data are not publicly available due to privacy or ethical restrictions.

## References

[1] D. R. Yao , I. Kim , S. Yin , and W. Gao , “Multimodal Soft Robotic Actuation and Locomotion,” Advanced Materials 36, no. 19 (2024): 2308829, 10.1002/adma.202308829. 38305065

[2] M. Li , A. Pal , A. Aghakhani , A. P. Francesch , and M. Sitti , “Soft Actuators for Real‐World Applications,” Nature Reviews Materials 7 (2022): 235–249, 10.1038/s41578-021-00389-7. PMC7612659 35474944

[3] A. Miriyev , “A Focus on Soft Actuators,” Actuators 8, no. 4 (2019): 74, 10.3390/act8040074.

[4] D. Rus and M. T. Tolley , “Design, Fabrication and Control of Soft Robots,” Nature 521 (2015): 467–475, 10.1038/nature14543. 26017446

[5] J. A. C. Liu , J. H. Gillen , S. R. Mishra , E. E. Evans , and J. B. Tracy , “Photothermally and Magnetically Controlled Reconfiguration of Polymer Composites for Soft Robotics,” Science Advances 5, no. 8 (2019): aaw2897, 10.1126/sciadv.aaw2897. PMC6677553 31414046

[6] J. Hu , Z. Nie , M. Wang , Z. Liu , S. Huang , and H. Yang , “Springtail‐Inspired Light‐Driven Soft Jumping Robots Based on Liquid Crystal Elastomers With Monolithic Three‐Leaf Panel Fold Structure,” Angewandte Chemie International Edition 62, no. 9 (2023): 202218227, 10.1002/anie.202218227. 36624053

[7] J. Gong , L. Hou , Y. C. Ching , K. Y. Ching , N. D. Hai , and C. H. Chuah , “A Review of Recent Advances of Cellulose‐Based Intelligent‐Responsive Hydrogels as Vehicles for Controllable Drug Delivery System,” International Journal of Biological Macromolecules 264, no. 2 (2024): 130525, 10.1016/j.ijbiomac.2024.130525. 38431004

[8] Y. Kim , G. A. parada , S. Liu , and X. Zhao , “Ferromagnetic Soft Continuum Robots,” Science Robotics 4, no. 33 (2019): aax7329, 10.1126/scirobotics.aax7329. 33137788

[9] X. Tang , H. Li , T. Ma , et al., “A Review of Soft Actuator Motion: Actuation, Design, Manufacturing and Applications,” Actuators 11, no. 11 (2022): 331, 10.3390/act11110331.

[10] X. Yang , Y. Sun , Y. Fu , et al., “Soft Actuators Based on Stimuli‐Responsive Materials: From Material Design to Robotic Systems,” Chemistry of Materials 38, no. 8 (2026): 3904–3930, 10.1021/acs.chemmater.6c00379.

[11] T. Wang , T. Jin , W. Lin , et al., “Multimodal Sensors Enabled Autonomous Soft Robotic System With Self‐Adaptive Manipulation,” ACS Nano 18, no. 14 (2024): 9980–9996, 10.1021/acsnano.3c11281. 38387068

[12] H. Wang , Z. Zhu , H. Jin , R. Wei , L. Bi , and W. Zhang , “Magnetic Soft Robots: Design, Actuation, and Function,” Journal of Alloys and Compounds 922 (2022): 166219, 10.1016/j.jallcom.2022.166219.

[13] M. Seong , K. Sun , S. Kim , et al., “Multifunctional Magnetic Muscles for Soft Robotics,” Nature Communications 15 (2024): 7929, 10.1038/s41467-024-52347-w. PMC11387479 39256389

[14] Y. Jung , K. Kwon , J. Lee , and S. Ho , “Untethered Soft Actuators for Soft Standalone Robotics,” Nature Communications 15 (2024): 3510, 10.1038/s41467-024-47639-0. PMC11045848 38664373

[15] F. Ahmed , M. Waqas , B. Jawed , et al., “Decade of Bio‐Inspired Soft Robots: A Review,” Smart Materials and Structures 31, no. 7 (2022): 073002, 10.1088/1361-665X/ac6e15.

[16] W. Hu , G. Z. Lum , M. Mastrangeli , and M. Sitti , “Small‐Scale Soft‐Bodied Robot With Multimodal Locomotion,” Nature 554 (2018): 81–85, 10.1038/nature25443. 29364873

[17] J. A. Carpenter , T. B. Eberle , S. Schuerle , A. Rafsanjani , and A. R. Studart , “Facile Manufacturing Route for Magneto‐Responsive Soft Actuators,” Advanced Intelligent Systems 3, no. 8 (2021): 2000283, 10.1002/aisy.202000283.

[18] X. Cao , S. Xuan , S. Sun , Z. Xu , J. Li , and X. Gong , “3D Printing Magnetic Actuators for Biomimetic Applications,” ACS Applied Materials & Interfaces 13, no. 25 (2021): 30127–30136, 10.1021/acsami.1c08252. 34137263

[19] Y. Kim , H. Yuk , R. Zhao , S. A. Chester , and X. Zhao , “Printing Ferromagnetic Domains for Untethered Fast‐Transforming Soft Materials,” Nature 558 (2018): 274–279, 10.1038/s41586-018-0185-0. 29899476

[20] Y. Ju , R. Hu , Y. Xie , et al., “Reconfigurable Magnetic Soft Robots With Multimodal Locomotion,” Nano Energy 87 (2021): 106169, 10.1016/j.nanoen.2021.106169.

[21] X. Wang , B. Han , R.‐P. Yu , et al., “Magnetic‐Responsive Fe_3_O_4_ Nanoparticle‐Impregnated Cellulose Paper Actuators,” Extreme Mechanics Letters 25 (2018): 53–59, 10.1016/j.eml.2018.10.003.

[22] X. Wang , G. Mao , J. Ge , et al., “Untethered and Ultrafast Soft‐Bodied Robots,” Communications Materials 1 (2020): 67, 10.1038/s43246-020-00067-1.

[23] X. Zhao , H. Yao , Y. Lv , et al., “Reprogrammable Magnetic Soft Actuators With Microfluidic Functional Modules via Pixel‐Assembly,” Small 20, no. 28 (2024): 2310009, 10.1002/smll.202310009. 38295155

[24] Y. Lee , F. Koehler , T. Dillon , et al., “Magnetically Actuated Fiber‐Based Soft Robots,” Advanced Materials 35, no. 38 (2023): 2301916, 10.1002/adma.202301916. PMC10526629 37269476

[25] H. Zhu , W. Song , H. Tian , et al., “Microstructure‐Enhanced Magnetic‐Driven Soft Actuator with High Force and Large Deformation,” ACS Applied Materials & Interfaces 17, no. 17 (2025): 55463–55476, 10.1021/acsami.5c14850. 40957725

[26] D. Tang , C. Zhang , H. Sun , et al., “Origami‐Inspired Magnetic‐Driven Soft Actuators With Programmable Designs and Multiple Applications,” Nano Energy 89 (2021): 106424, 10.1016/j.nanoen.2021.106424.

[27] Y. Yang , C. AI , W. Chen , J. Zhen , X. Kong , and Y. Jiang , “Recent Advances in Sources of Bio‐Inspiration and Materials for Robotics and Actuators,” Small Methods 7, no. 9 (2023): 2300338, 10.1002/smtd.202300338. 37381685

[28] H. W. Krenn , “Feeding Mechanisms of Adult Lepidoptera: Structure, Function, and Evolution of the Mouthparts,” Annual Review of Entomology 55 (2010): 307–327, 10.1146/annurev-ento-112408-085338. PMC4040413 19961330

[29] J. A. H. P. Sol , A. R. Peeketi , N. Vyas , A. P. H. J. Schenning , R. K. Annabattula , and M. G. Debije , “Butterfly Proboscis‐Inspired Tight Rolling Tapered Soft Actuators,” Chemical Communications 55, no. 12 (2019): 1726–1729, 10.1039/c8cc09915d. 30644478

[30] P.‐W. Lin and C.‐H. Liu , “Bio‐Inspired Soft Proboscis Actuator Driven by Dielectric Elastomer Fluid Transducers,” Polymers 11, no. 1 (2019): 142, 10.3390/polym11010142. 30960125 PMC6401884

[31] Z. Burghard , A. Leineweber , P. A. van Aken , T. Dufaux , M. Burghard , and J. Bill , “Hydrogen‐Bond Reinforced Vanadia Nanofiber Paper of High Stiffness,” Advanced Materials 25, no. 17 (2013): 2468–2473, 10.1002/adma.201300135. 23468458

[32] A. Knöller , T. Runčevski , R. E. Dinnebier , J. Bill , and Z. Burghard , “Cuttlebone‐Like V_2_O_5_ Nanofibre Scaffolds – Advances in Structuring Cellular Solids,” Scientific Reports 7 (2017): 42951, 10.1038/srep42951. 28218301 PMC5317173

[33] B. Wicklein , A. M. Diem , A. Knöller , et al., “Electrochromism: Dual‐Fiber Approach Toward Flexible Multifunctional Hybrid Materials,” Advanced Functional Materials 28, no. 27 (2018): 1870186, 10.1002/adfm.201870186.

[34] M. Richter , J. Sikorski , P. Makushko , et al., “Locally Addressable Energy Efficient Actuation of Magnetic Soft Actuator Array Systems,” Advanced Sciences 10, no. 24 (2023): 2302077, 10.1002/advs.202302077. PMC10460866 37330643

[35] M. L. Dezaki and M. Bodaghi , “Magnetically Controlled Bio‐Inspired Elastomeric Actuators With High Mechanical Energy Storage,” Soft Matter 19, no. 16 (2023): 3015–3032, 10.1039/D3SM00266G. 37021651

[36] J. Livage , “Vanadium Pentoxide Gels,” Chemistry of Materials 3, no. 4 (1991): 578–593, 10.1021/cm00016a006.

[37] S. Park and K. Schulten , “Calculating Potentials of Mean Force From Steered Molecular Dynamics Simulations,” Journal of Chemical Physics 120, no. 13 (2004): 5946–5961, 10.1063/1.1651473. 15267476

[38] M. D. Nguyen , H. V. Tran , S. Xu , and T. R. Lee , “Fe_3_O_4_ Nanoparticles: Structures, Synthesis, Magnetic Properties, Surface Functionalization, and Emerging Applications,” Applied Sciences 11, no. 23 (2021): 11301, 10.3390/app112311301. 35844268 PMC9285867

[39] A. L. Andrade , J. D. Fabris , J. D. Ardisson , M. A. Valente , and J. M. Ferreira , “Effect of Tetramethylammonium Hydroxide on Nucleation, Surface Modification and Growth of Magnetic Nanoparticles,” Journal of Nanomaterials 2012, no. 1 (2012): 454759, 10.1155/2012/454759.

[40] W. Wu , Q. He , and C. Jiang , “Magnetic Iron Oxide Nanoparticles: Synthesis and Surface Functionalization Strategies,” Nanoscale Research Letters 3 (2008): 397, 10.1007/s11671-008-9174-9. 21749733 PMC3244954

[41] Y. Kim and X. Zhao , “Magnetic Soft Materials and Robots,” Chemical Reviews 122, no. 5 (2022): 5317, 10.1021/acs.chemrev.1c00481. 35104403 PMC9211764

[42] L. H. Gaabour , “Analysis of Spectroscopic, Optical and Magnetic Behaviour of PVDF/PMMA Blend Embedded by Magnetite (Fe_3_O_4_) Nanoparticles,” Optics and Photonics Journal 10, no. 8 (2020): 197–209, 10.4236/opj.2020.108021.

[43] A. M. Diem , A. Knöller , Z. Burghard , and J. Bill , “Free‐Standing Nanostructured Vanadium Pentoxide Films for Metal‐Ion Batteries,” Nanoscale 10, no. 33 (2018): 15736–15746, 10.1039/c8nr04033h. 30094430

[44] J. Wang , Q. Cheng , and Z. Tang , “Layered Nanocomposites Inspired by the Structure and Mechanical Properties of Nacre,” Chemical Society Reviews 41, no. 3 (2012): 1111–1129, 10.1039/c1cs15106a. 21959863

[45] M. E. Launey and R. O. Ritchie , “On the Fracture Toughness of Advanced Materials,” Advanced Materials 21, no. 20 (2009): 2103–2110, 10.1002/adma.200803322.

[46] J. Wang , Q. Cheng , L. Lin , and L. Jiang , “Synergistic Toughening of Bioinspired Poly(vinyl alcohol)–Clay–Nanofibrillar Cellulose Artificial Nacre,” ACS Nano 8, no. 3 (2014): 2739–2745, 10.1021/nn406428n. 24506706

[47] A. M. Diem , J. Bill , and Z. Burghard , “Fast One‐Step Fabrication of Highly Regular Microscrolls With Controllable Surface Morphology,” Advanced Science 10, no. 10 (2023): 2302103, 10.1002/advs.202302103. 37162217 PMC10375128

[48] S. P. Timoshenko and J. M. Gere , “Chapter 2‐Elastic Buckling of Bars and Frames,” in Theory of Elastic Stability, 2nd ed. (Dover Publications Inc., 2012), ISBN‐10: 0‐486‐47207‐8.

[49] T. Xu , J. Zhang , M. Salehizadeh , O. Onaizah , and E. Diller , “Millimeter‐Scale Flexible Robots With Programmable Three‐Dimensional Magnetization and Motions,” Science Robotics 4, no. 29 (2019): aav4494, 10.1126/scirobotics.aav4494. 33137716

[50] A. Benassi , J. Schwenk , M. A. Marioni , H. J. Hug , and D. Passerone , “Microscale Motion Control Through Ferromagnetic Films,” Advanced Materials Interfaces 1, no. 4 (2014): 1400023, 10.1002/admi.201400023.

[51] J. Miao and S. Sun , “Design, Actuation, and Functionalization of Untethered Soft Magnetic Robots With Life‐Like Motions: A Review,” Journal of Magnetism and Magnetic Materials 586 (2023): 171160, 10.1016/j.jmmm.2023.171160.

[52] L. Shui , L. Zhu , Z. Yang , Y. Liu , and X. Chen , “Energy Efficiency of Mobile Soft Robots,” Soft Matter 13, no. 44 (2017): 8223–8233, 10.1039/c7sm01617d. 29083008

