# Supplementary material for: Mechanically Assisted Magnetic Actuation in Ceramic‐Based Microscrolls for Fast and Durable Soft Robotic Systems
Source: Adv Mater. 2026 Aug 8;38(52):e74544. doi: 10.1002/adma.74544 (PMC13579072; doi:10.1002/adma.74544)
Supplement: Supplementary file 1 [file ADMA-38-e74544-s002.pdf]

Supporting Information

Mechanically Assisted Magnetic Actuation in Ceramic-Based Microscrolls for Fast and Durable Soft Robotic Systems

*Semi Kim, Shravan R. Kousik, Petia Atanasova, Eberhard Goering, Joachim Bill, Zaklina Burghard**

SI 1. Additional structural, compositional, and magnetic characterization

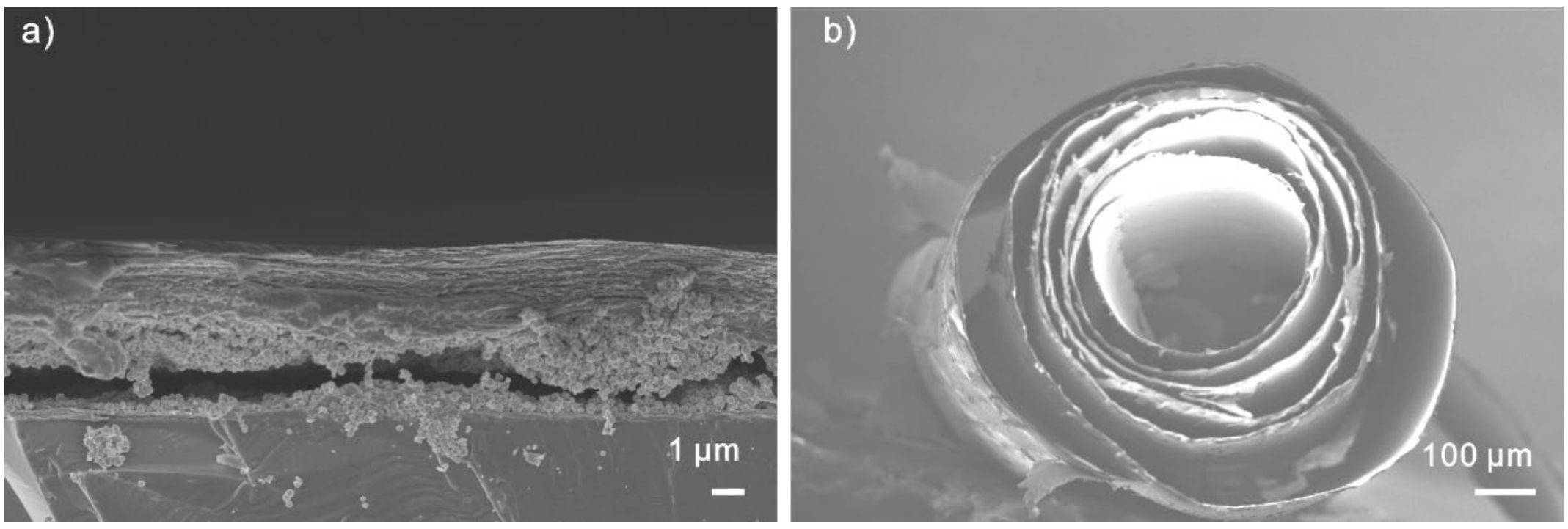

Figure S1. Cross-sectional morphology of hybrid films prepared without TEAOH and the corresponding microscroll. (a) Cross-sectional SEM image of a VNF/Fe_3O_4 hybrid film containing 8.6 wt% nanoparticles prepared without TEAOH treatment, showing irregular interlayer spacing, non-uniform film thickness, and pronounced nanoparticle agglomeration with local voids formed during fracture. (b) Cross-sectional SEM image of a microscroll (2 mm width, 10 mm scrolling length) fabricated from the same TEAOH-free hybrid film. The lamellar spacing within the scroll is irregular, reflecting the poor nanoparticle dispersion and structural non-uniformity of the precursor film. These observations confirm that TEAOH treatment enhances colloidal stability of Fe_3O_4 nanoparticles and promotes their more homogeneous incorporation within the VNF layered structure.

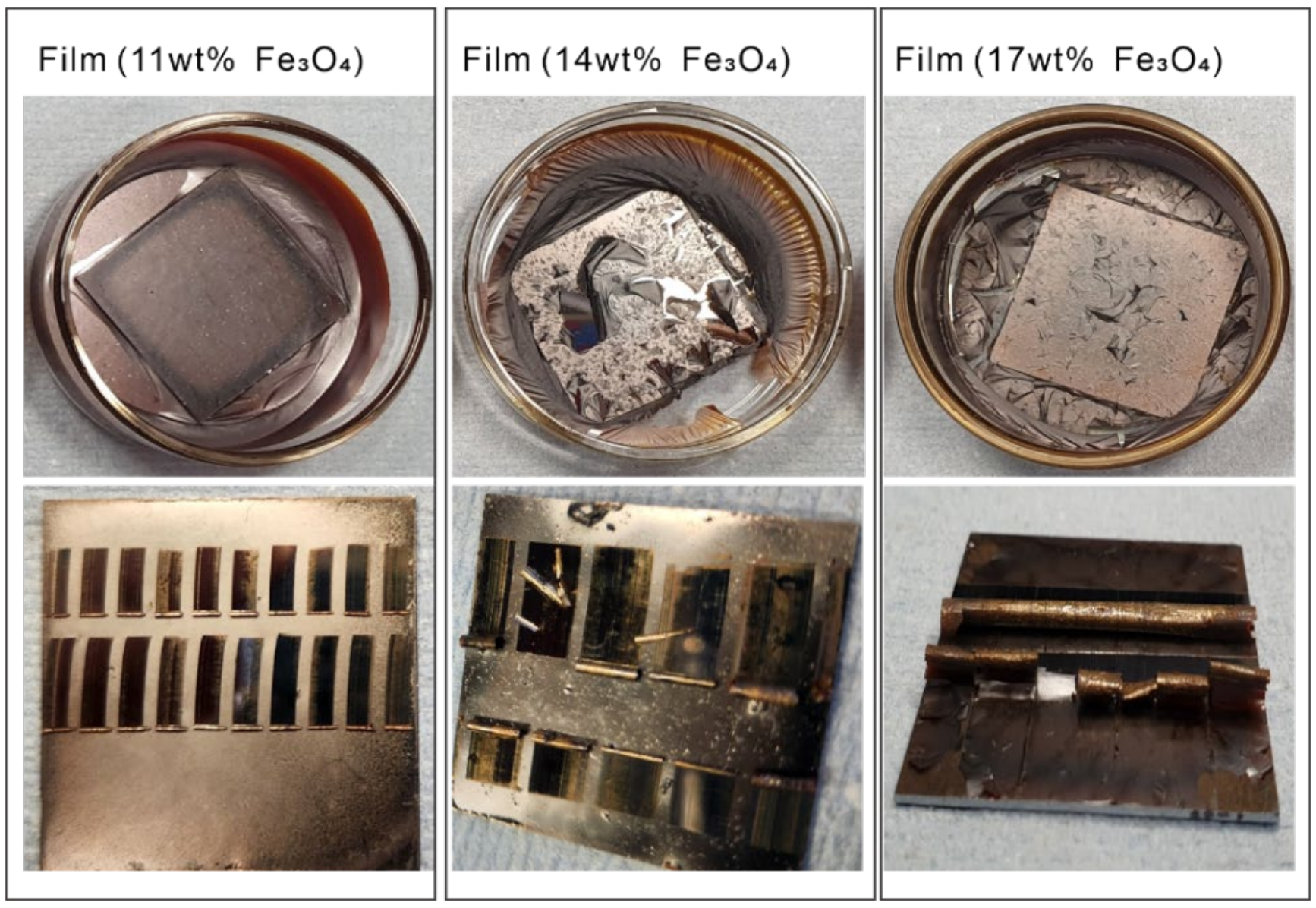

Figure S2. Influence of Fe_3O_4 nanoparticle loading on hybrid film formation and scrollability. Digital images of corresponding dried films. Films containing up to 11 wt% Fe_3O_4 exhibited uniform morphology and retained mechanical integrity upon drying, enabling reproducible scrolling; the representative case of 11 wt% Fe_3O_4 is shown. In contrast, films prepared with Fe_3O_4 contents > 11 wt% displayed severe cracking, delamination, and loss of mechanical integrity during drying, preventing successful microscroll formation.

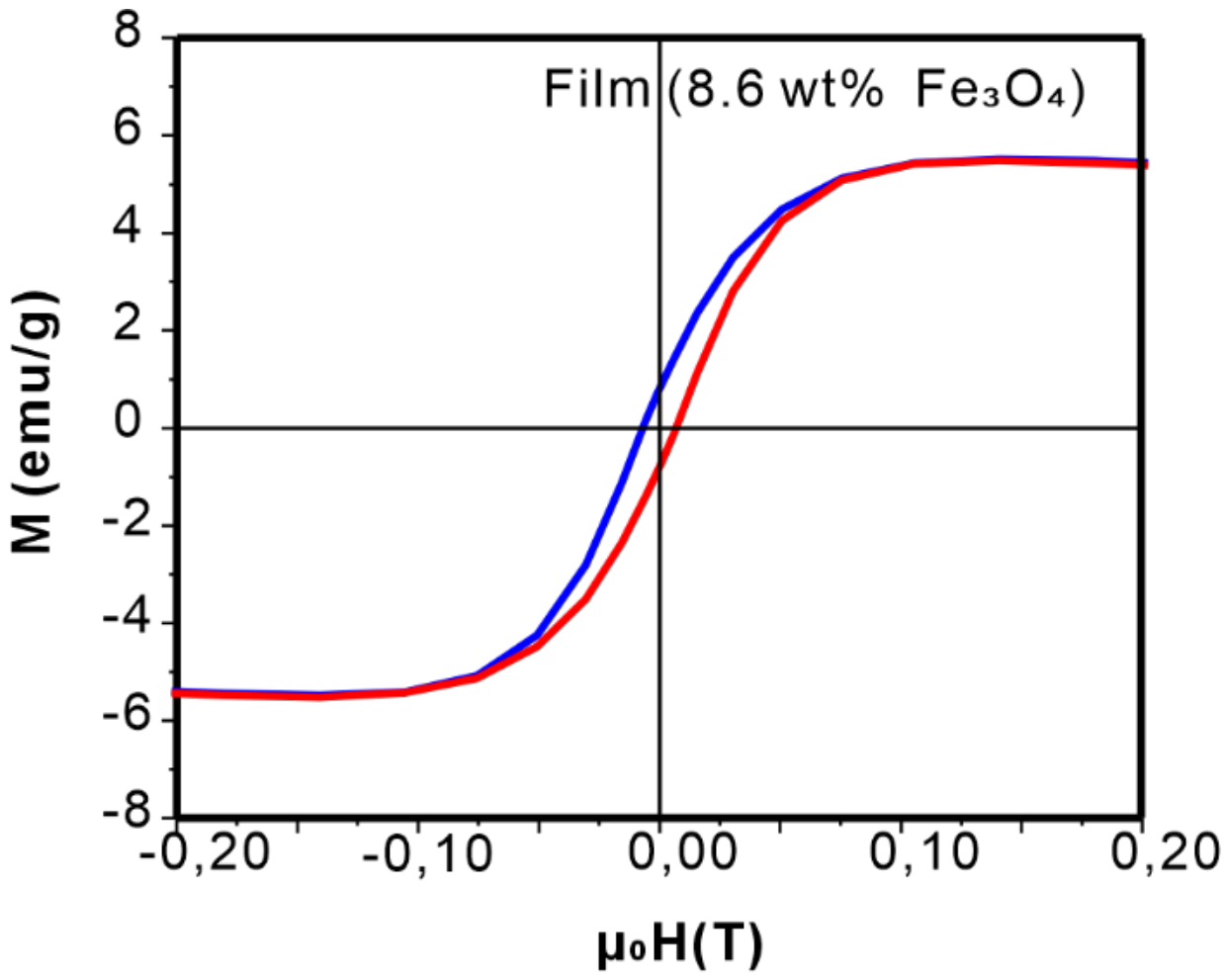

Figure S3. Magnetization curve of a hybrid film containing 8.6 wt% Fe_3O_4 at room temperature. Forward (blue) and reverse (red) M–H scans show superparamagnetic-like behavior with small coercive fields, consistent with primary particle sizes of 10 – 20 nm. The lower saturation magnetization reflects the reduced magnetic fraction and dispersion of nanoparticles within the VNF matrix. The saturation magnetization of the hybrid film is 6.47 emu/g.

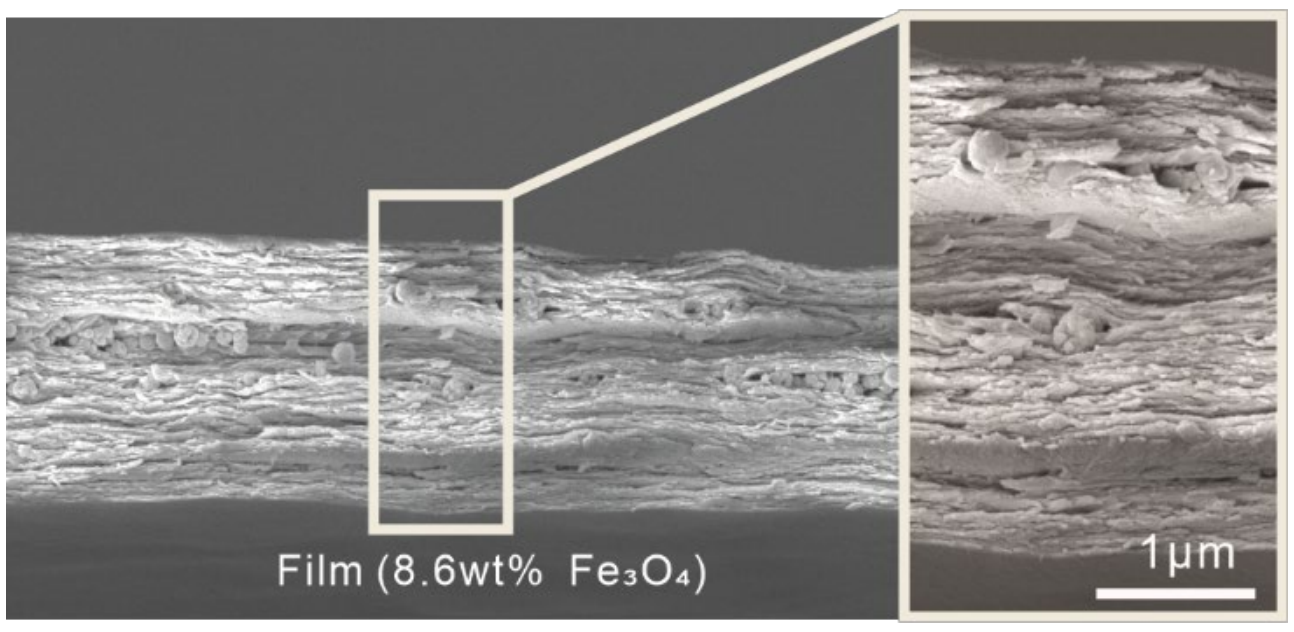

Figure S4. Cross-sectional SEM images of fractured hybrid films containing 8.6 wt% Fe_3O_4, showing multilayered fracture morphology with nanoparticle-rich domains embedded within the VNF lamellar structure.

SI 2. Microstructural deformation and energy-dissipation mechanism under tensile loading

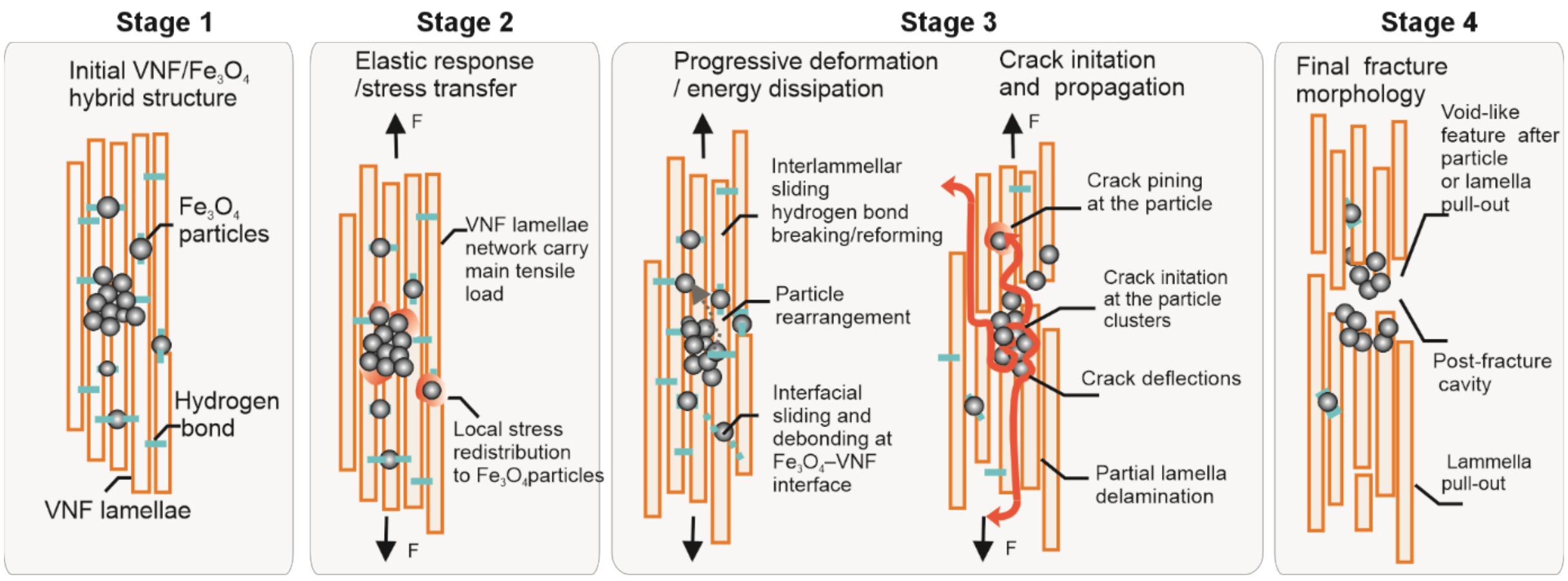

Figure S5. Proposed deformation and energy-dissipation mechanism of VNF/Fe_3O_4 hybrid films under tensile loading.

Stage 1 illustrates the initial hybrid architecture, consisting of a continuous VNF lamellar network containing embedded Fe_3O_4 particles and particle-rich regions. Stage 2 depicts the elastic response, in which the VNF lamellae form the primary load-bearing scaffold and carry most of the tensile load, while Fe_3O_4 particles locally perturb the stress field but do not form a continuous reinforcing framework.

With increasing strain progressive deformation and energy dissipation occur through interlamellar sliding, partial delamination of VNF lamella, and limited frictional sliding at VNF–VNF and VNF–Fe_3O_4 interfaces (Stage 3). Crack initiation is proposed to occur preferentially in particle-rich regions, where local stress concentrations develop. During subsequent propagation, the crack is repeatedly deflected and pinned by Fe_3O_4-rich regions and is therefore forced to follow a more tortuous path along and across the VNF lamellae rather than propagating straight through the film.

At maximum strain, crack propagation continues through the lamellar architecture, accompanied by lamella pull-out, local debonding of particle rich domains, and post-fracture cavity formation, which give rise to void-like features on the fracture surface (Stage 4). These void-like features are interpreted as post-fracture pull-out/debonding sites rather than pre-existing pores in the intact hybrid film. Together, the mechanisms summarized in Figure S5 explain how the hybrid films maintain the high stiffness of the VNF scaffold while exhibiting increased fracture energy and improved mechanical robustness.

SI 3. Geometry control and parallel fabrication of microscrolls

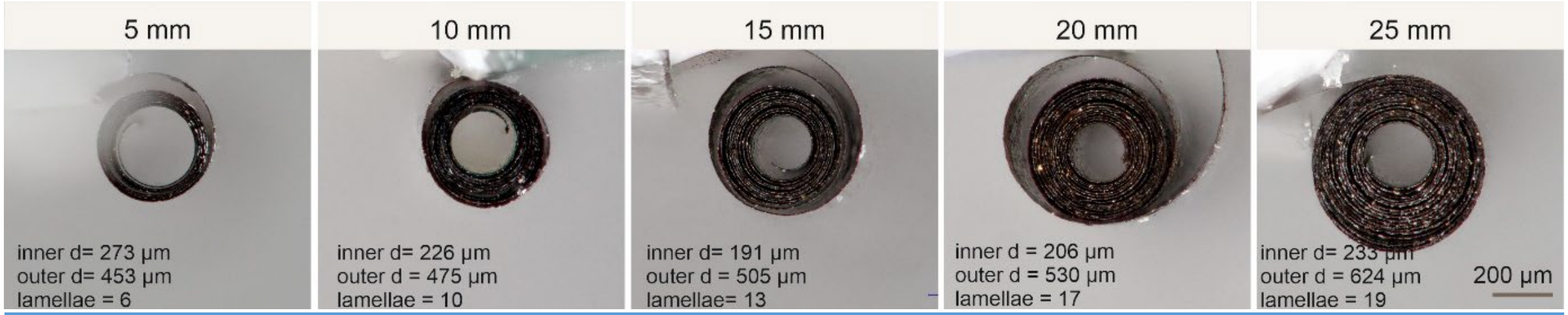

Figure S6. Geometry of as-prepared VNF/Fe_3O_4 microscrolls with different scrolling lengths. Optical cross-sectional images of as-prepared microscrolls obtained from rolling lengths of 5, 10, 15, 20, and 25 mm. The number of lamellae increases from approximately 6 to 19 with increasing rolling length, while the outer diameter increases from 453 to 624 µm. These geometrical parameters define the curvature-dependent elastic resistance and interlamellar locking that govern the actuation threshold and cascade unrolling behavior.

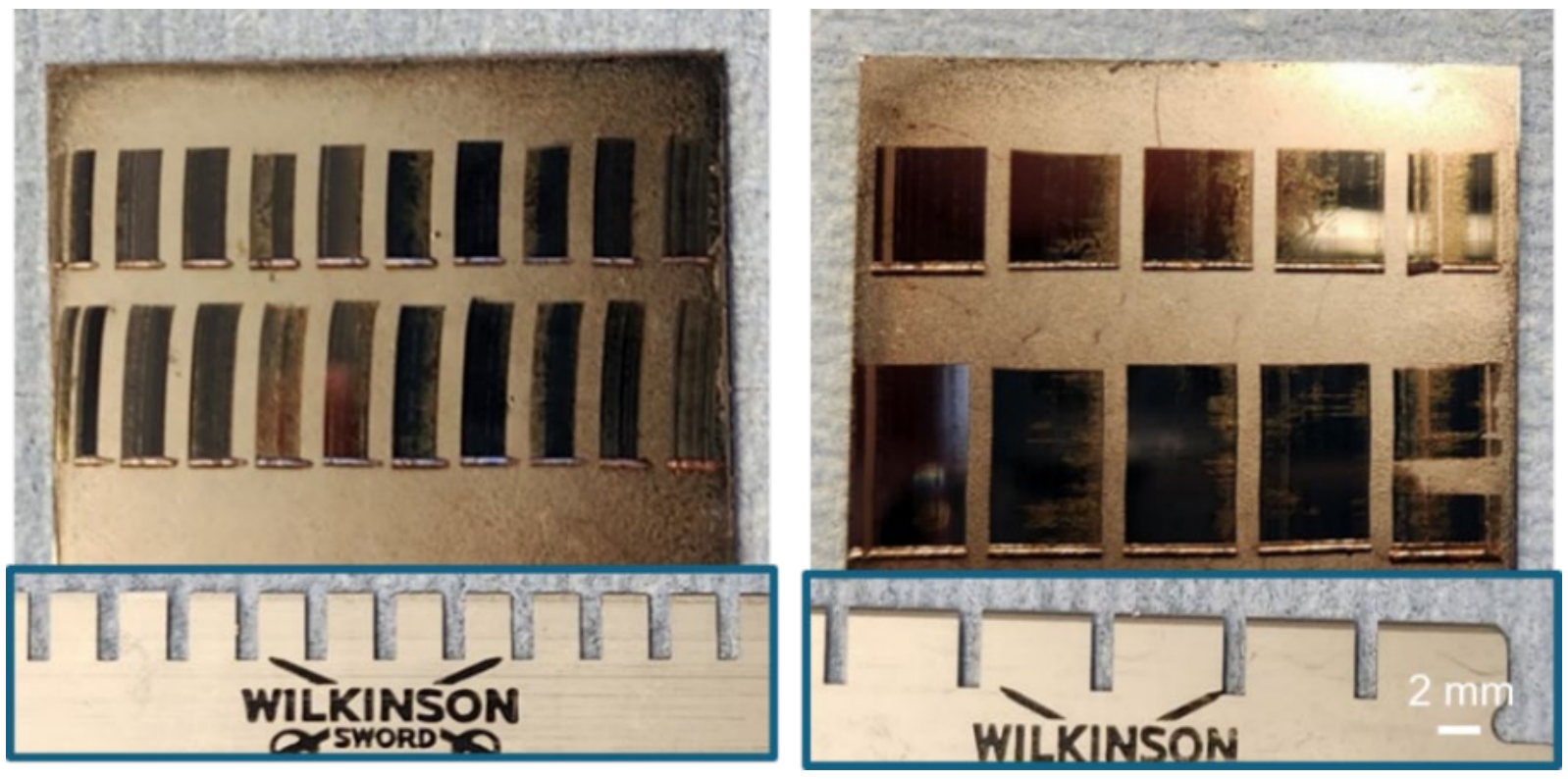

Figure S7. Parallel fabrication of microscrolls using comb-geometry razor blades. Digital images of two custom razor blades with defined cutting widths (2 mm and 5 mm). The 2 mm blade enables simultaneous fabrication of multiple narrow microscrolls on silicon substrates (left), while the 5 mm blade produces wider individual scrolls (right), demonstrating scalable scroll fabrication.

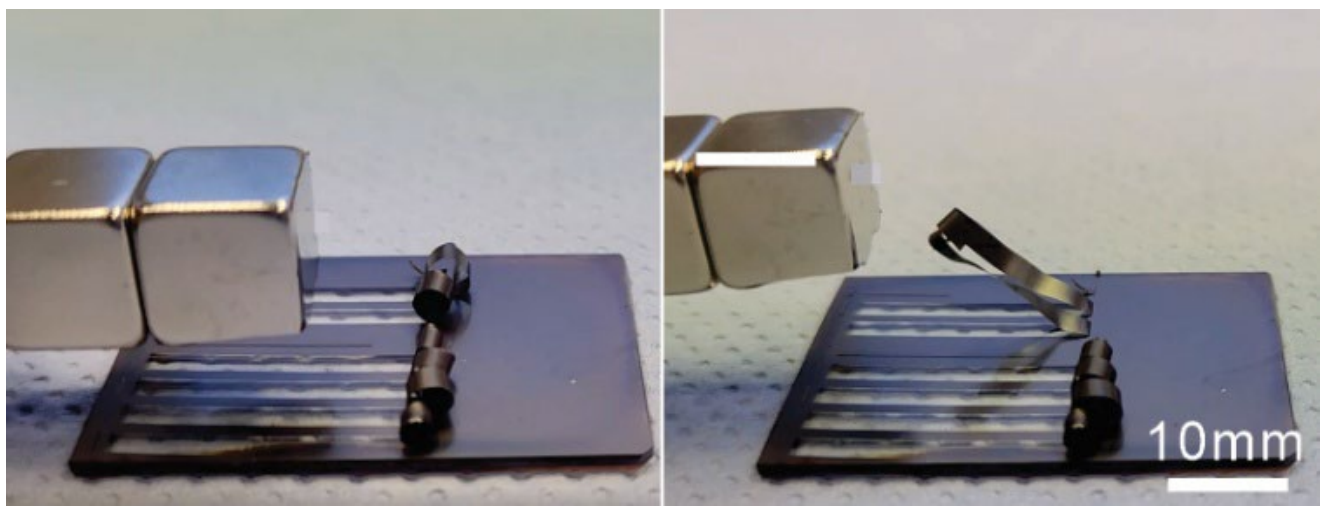

Figure S8. Digital images showing actuation at distances > 7 mm when four permanent magnets are used, enabling simultaneous multi-scroll response.

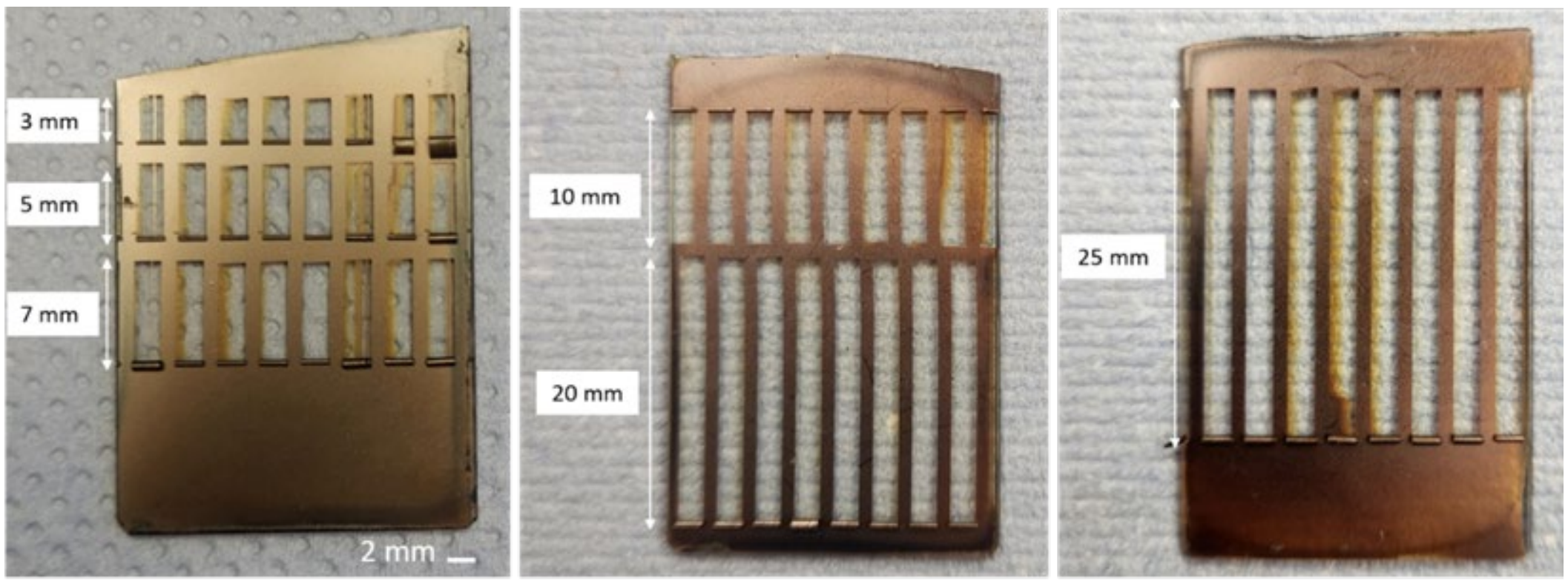

Figure S9. Digital images of microscrolls fabricated on glass substrates, demonstrating varying scroll lengths ranging from 3 mm to 25 mm. Uniform rolling behavior is maintained across the full range of geometries.

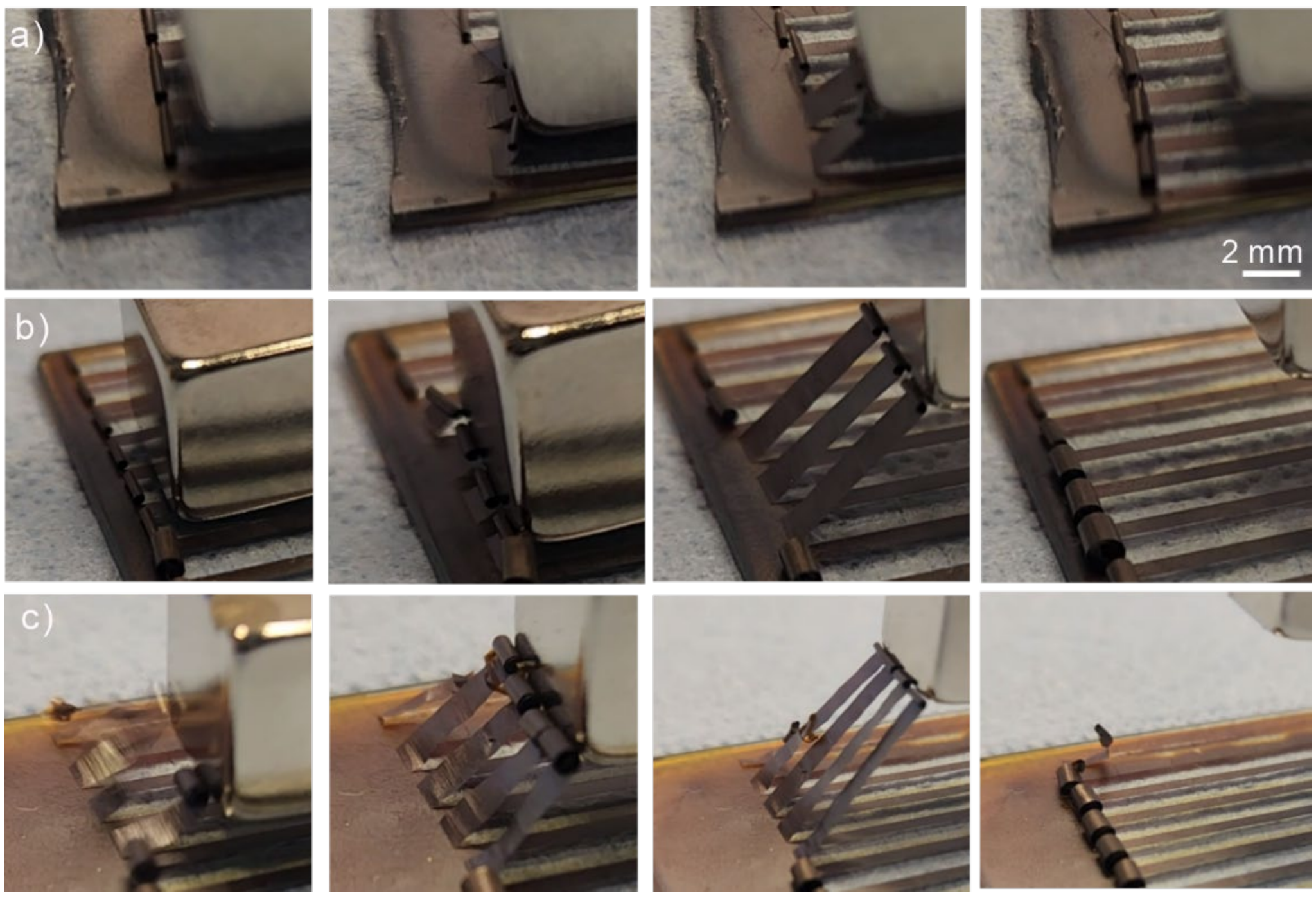

Figure S10. Digital images showing magnetic actuation behaviors of VNF/Fe_3O_4 microscrolls with different scrolling lengths. (a) 10 mm, (b) 20 mm, and (c) 25 mm. Shorter scrolls exhibit higher actuation thresholds due to smaller internal radii and stronger interlamellar coupling, whereas longer scrolls unroll only partially because their outer lamellae enter regions of weaker magnetic field. These observations are consistent with the geometry-dependent actuation limits discussed in Section 2.5 of the main text.

SI 4. SQUID magnetometry and magnetic torque estimation

To quantify the magnetic contribution to the dual magneto-mechanical actuation mechanism, SQUID magnetometry was performed on individual 15 mm VNF/Fe_3O_4 microscrolls. A representative magnetization curve is shown in Figure S11.

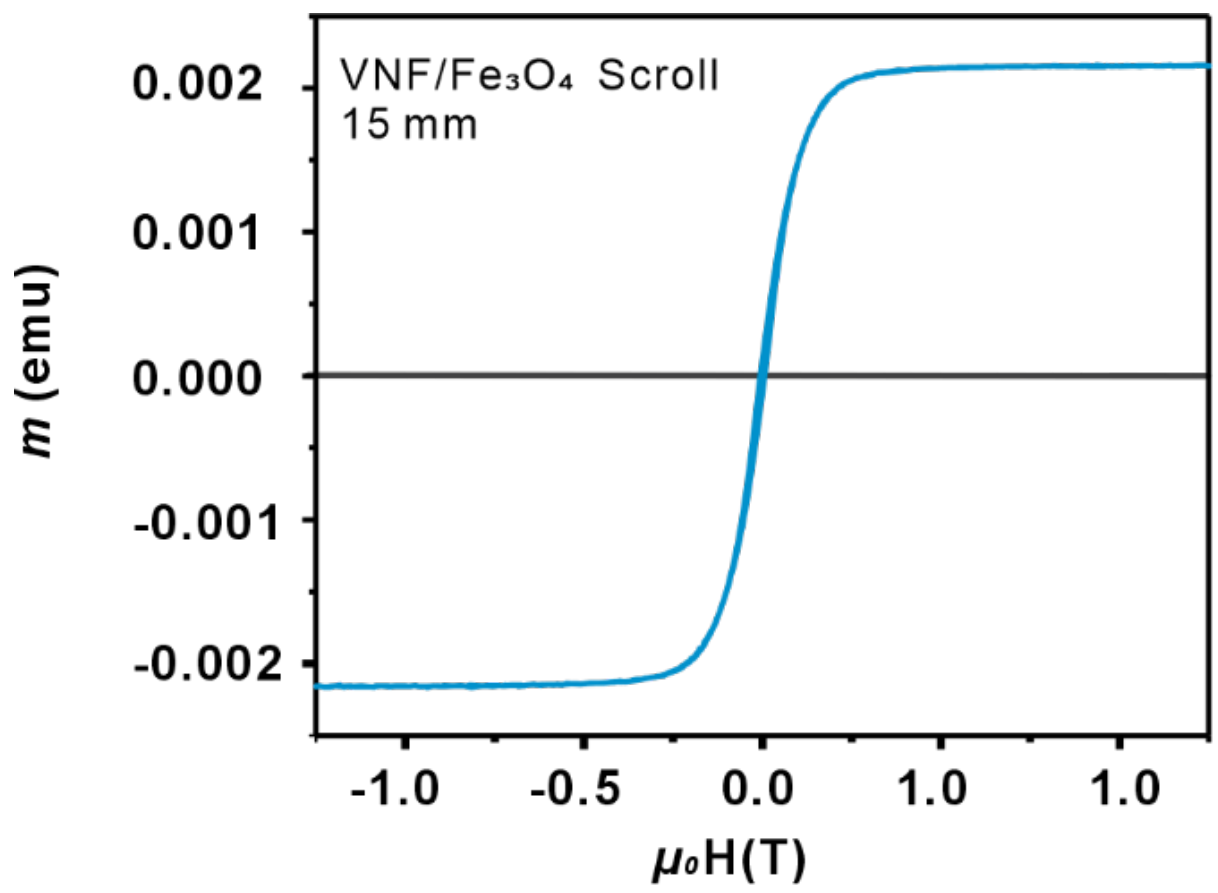

Figure S11. SQUID magnetometry of an individual 15 mm VNF/Fe_3O_4 microscroll. The measurement shows near-zero remanent magnetization and approach to saturation at high magnetic field, confirming the field-induced magnetic response of the scroll.

The SQUID measurements show near-zero remanent magnetization under zero applied field, consistent with the partially blocked superparamagnetic character of the Fe_3O_4 nanoparticles discussed in Section 2.2. The magnetic moment increases rapidly at low fields and approaches a near-saturation plateau at higher fields, giving an estimated saturation moment of:

$$m_{sat} = 2.157 \times 10^{-3} \text{ emu} = 2.157 \times 10^{-6} \text{ Am}^2$$

The magnetic torque was estimated from the measured magnetic field profile of the magnet and the SQUID-derived magnetic moment of the scroll. Because the magnetic moment is not fully saturated at all field values relevant to actuation, the field-dependent magnetic moment was approximated as:

$$m(B) = m_{sat} f(B)$$

with:

$$f(B) = tanh(\frac{B}{B_0})$$

where f(B) is the fractional saturation and B_0 is a characteristic field parameter. The value of B_0 was determined from the normalized SQUID M–H curve by imposing:

$$\frac{m(60mT)}{m_{sat}} = 0.85$$

which gives:

$$B_0 \approx 47.8 \text{ mT}$$

The magnetic torque as a function of distance from the magnet edge was calculated as:

$$\tau_m(d) = m(B(d))B(d)$$

where B(d) is the local magnetic field measured by Hall-probe measurements as a function of distance from the magnet edge (Figure 4b main text). The calculated values are summarized in Table S1.

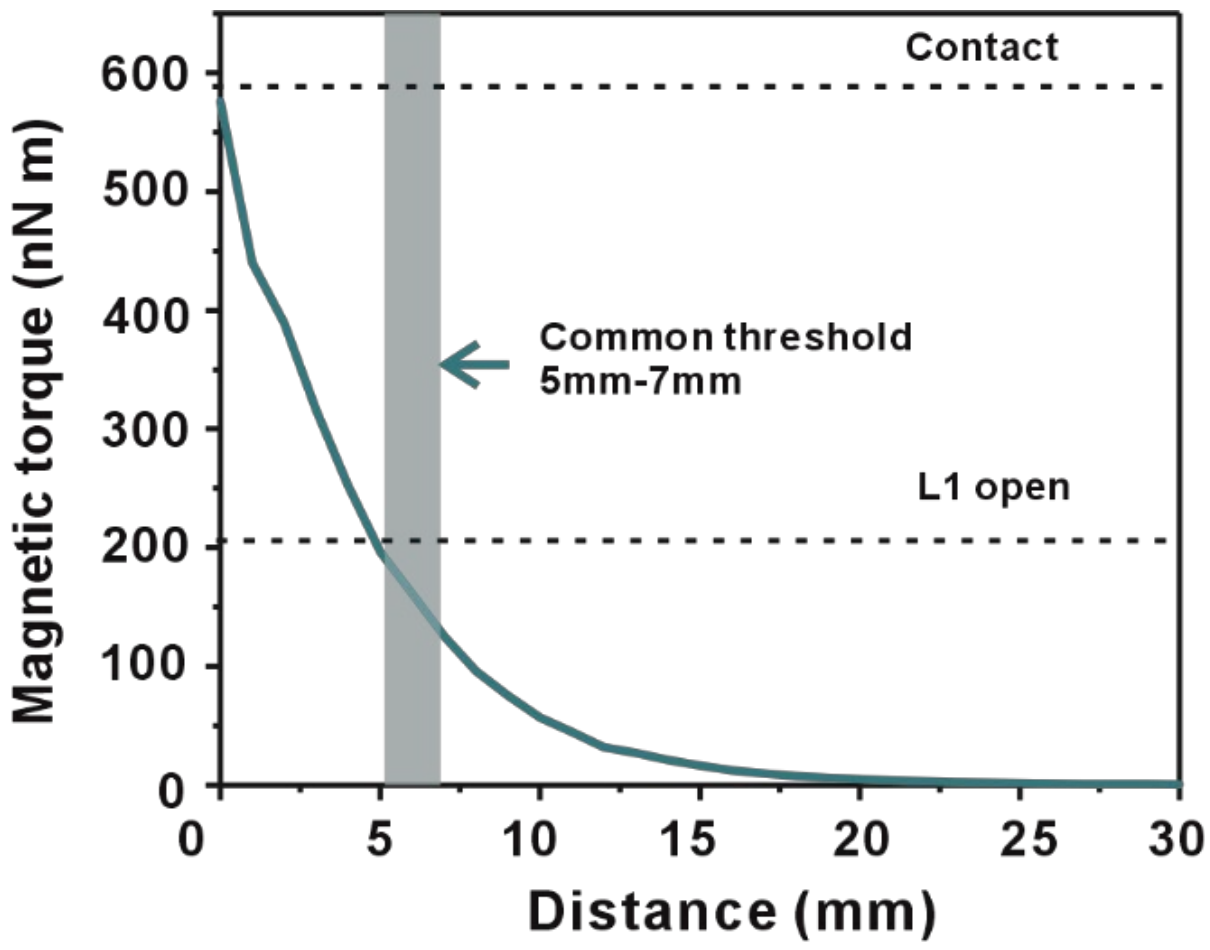

Figure S12. Magnetic field profile and SQUID-derived magnetic torque as a function of distance from the magnet edge. Calculated magnetic torque $\tau_m(d)$ for a 15 mm VNF/Fe_3O_4 microscroll using the SQUID-derived field-dependent magnetic moment.

Table S1. SQUID-derived magnetic torque of the 15 mm VNF/Fe_3O_4 microscroll as a function of distance from the magnet edge.

Distance from magnet edge (mm)	B (mT)	*f*(B)	τ_m, (nN m)
0	266.8	1.00	576.6
5	94.6	0.96	196.4
8	54.6	0.81	96.0
10	39.1	0.67	56.9
15	17.8	0.36	16.7
30	4.2	0.09	0.78

For the microscrolls tested here, magnetic unrolling was commonly initiated when the magnet was positioned approximately 5–7 mm from the scroll edge. This distance range is treated as an experimentally relevant onset window rather than a universal threshold, because the apparent threshold depends on scroll geometry, packing density, and the degree of stored elastic strain. Over this onset window, the local magnetic field is approximately 60–95 mT, giving a magnetic torque of approximately:

$$\tau_{m,onset} \approx 110 - 196 \text{ nN m}$$

At direct magnet contact, the magnetic torque reaches:

$$\tau_{m,max} \approx 576 \text{ nN m}$$

These values define the magnetic torque available during the initiation and contact stages of actuation.

SI 5. Mechanical opening and analytical bending model

To independently quantify the mechanical component of the dual mechanism, mechanical opening tests were performed on a 15 mm VNF/Fe_3O_4 microscroll, and combined with an analytical restoring-force estimate based on the measured scroll geometry (Figure S13).

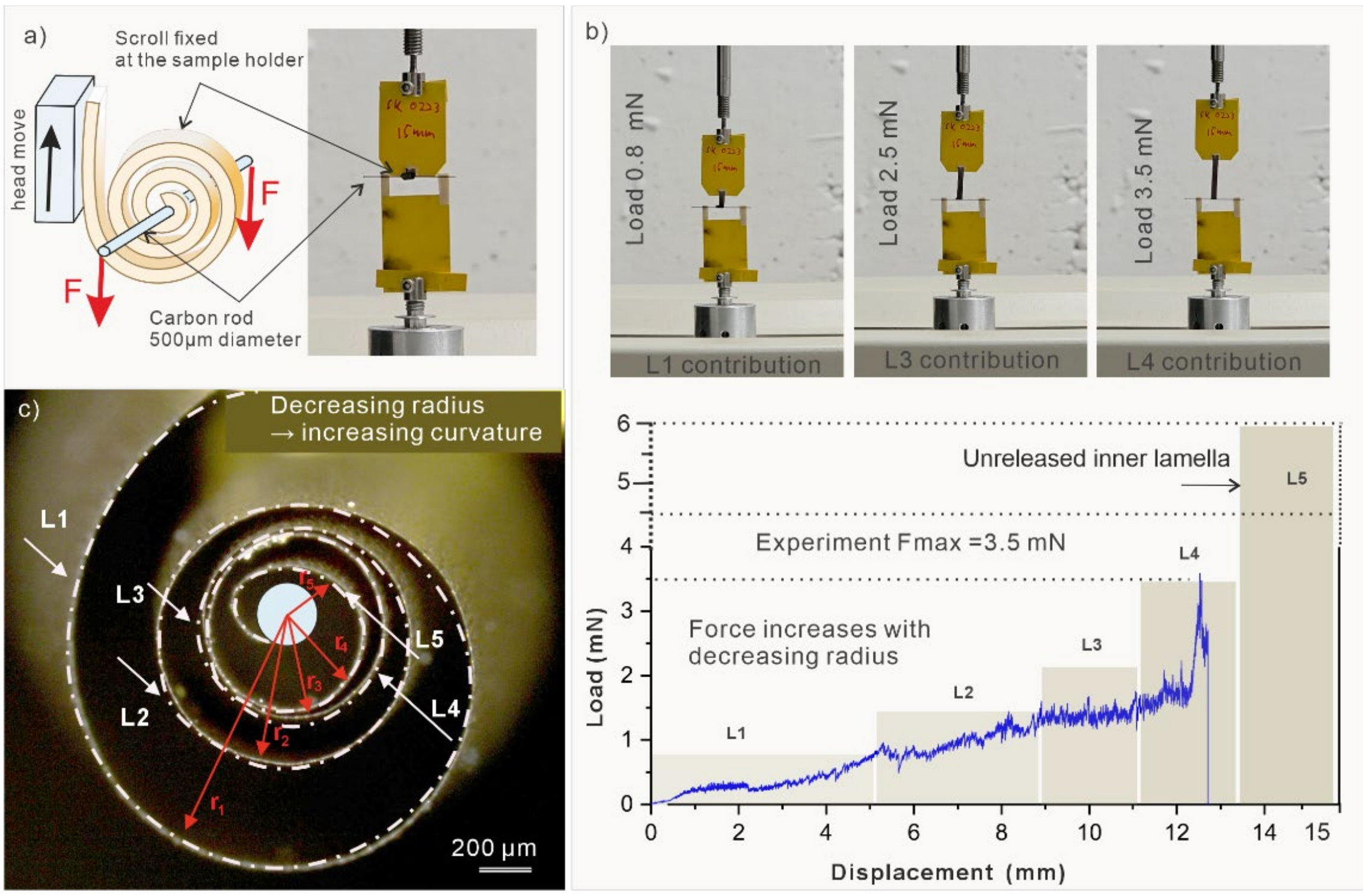

Figure S13. Quantitative mechanical analysis of scroll unrolling via tensile opening tests and analytical bending model. (a) Schematic illustration of the mechanical opening experiment. (b) Experimental force–displacement curve obtained during mechanical opening (Video S5), with shaded regions indicating progressive activation of lamellar segments L1–L5. The experimentally measured peak load reaches ~3.5 mN before slip occurs, whereas the analytical model predicts a cumulative restoring force of ~6.0 mN for complete engagement of all modeled lamellae, including the innermost segment. (c) Optical cross-section of the tested 15 mm VNF/Fe_3O_4 microscroll showing the multilamellar geometry used for the analytical model.

A carbon rod was inserted into the central opening of a 15 mm long scroll, while the outer end of the scroll was fixed (Figure S13 a). The scroll was pulled upward at a constant rate, while the carbon rod remained fixed in place. The resulting force–displacement response provides a

direct mechanical probe of the stored elastic resistance and interlamellar coupling within the multilamellar scroll (Figure S13 b). The force increased smoothly and continuously to a peak value of approximately 3.5 mN, without discrete force plateaus or sawtooth-like features that would be expected if individual lamellae detached independently. Optical snapshots recorded during the test confirmed that the scroll opened progressively and cohesively, with successive lamellar segments becoming engaged as the displacement increased.

To quantitatively assess the contribution of stored elastic strain energy to microscroll unrolling, we used an analytical bending model [41] based on the measured cross-sectional geometry of the same 15 mm VNF/Fe_3O_4 scroll used in the tensile opening experiment. The purpose of this model is not to provide a full finite-element description of the scroll, but to estimate the order of magnitude of the elastic restoring force associated with bending of the multilamellar structure.

The scroll was approximated as an Archimedean-like spiral composed of multiple lamellar segments. Each segment was defined by an effective radius r_i extracted from the optical cross-section of the tested scroll (Figure S13c). The selected radii were r_1=840 µm, r_2=580 µm, r_3=400 µm, r_4=360 µm, and r_5=260 µm.

The corresponding segment length was approximated as:

$$L_i = 2\pi r_i$$

The elastic bending energy stored in each curved lamellar segment is calculated using classical thin-plate bending theory:

$$E_{e,i} = \frac{1}{2} \mathrm{D} \kappa_i^2 \mathrm{A}_i$$

Here $E_{e,i}$ is the elastic bending energy stored in lamellar segment *i*, D is the bending stiffness of the film, $\kappa_i = 1/r_i$ is the curvature of the segment, and $A_i = wL_i$ is the area of the lamellar segment. The scroll width was w= 2mm. The bending stiffness was calculated as:

$$D = \frac{Eh^3}{12(1-\nu^2)}$$

Where E= 29 GPa is the Young's modulus of the 11 wt% VNF/Fe_3O_4 hybrid film, h= 4.2 µm is the film thickness, and υ = 0.1 is the Poisson's ratio assumed for ceramic-based hybrid film.

For each lamellar segment, the segmental restoring-force contribution was estimated by normalizing the bending energy of that segment by its effective segment length:

$$F_i \approx \frac{E_{ei}}{L_i}$$

The cumulative modeled restoring force was then obtained by summing the segmental contributions:

$$F_{cum} = \sum_i F_i$$

This segmental approach reflects the progressive activation of lamellae during tensile opening. Because F_i scales with $1/r_i^2$, inner lamellae with smaller radii contribute more strongly to the total modeled resistance.

Using the measured effective radii, the total stored bending energy was estimated as:

$$E_{e,total} = \sum_i E_{e,i} \approx 13.7\ \mu J$$

and the cumulative modeled restoring force for complete activation of all modeled lamellae was:

$$F_{cum,max} \approx 6.0 mN$$

This value is larger than the maximum force reached during the mechanical opening test, indicating that the experiment activated a substantial fraction of the stored elastic resistance but did not fully release the innermost lamella. Video analysis (Video S5, Supporting Information) shows that the scroll slipped from the carbon rod near the peak load, preventing complete activation of the final inner lamella. Therefore, the measured force corresponds to partial activation of the multilamellar structure, whereas the modeled $F_{cum,max}$ represents an idealized estimate for full engagement of all modeled lamellae.

Table S2. Estimated elastic bending energy and restoring-force contributions for the lamellar segments of the 15 mm VNF/Fe_3O_4 microscroll used in the tensile opening test.

Segment	r_i (μm)	$L_i=2\pi r_i$ (mm)	$E_{e,i}$ (μJ)	$F_i=E_{e,i}/L_i$ (mN)	Cumulative F_{cum}(mN)
L1	840	5.28	1.35	0.26	0.26
L2	580	3.64	1.96	0.54	0.79
L3	400	2.51	2.84	1.13	1.91
L4	360	2.26	3.16	1.40	3.32
L5	260	1.63	4.37	2.68	6.00

The model reproduces two key features of the mechanical opening experiment:
- the restoring-force contribution increases as the effective lamellar radius decreases, and
- the calculated force lies in the mN range, consistent with the measured peak load.

This supports the conclusion that stored elastic strain energy provides a mechanically significant contribution to the scroll unrolling.

To compare the elastic resistance with the SQUID-derived magnetic torque, the segmental restoring forces were converted into elastic torques according to:

$$\tau_{e,i} = F_i\, r_i$$

The cumulative elastic torque was obtained by summing the segmental torque contributions:

$$\tau_{e,cum} = \sum_i \tau_{e,i}$$

Table S3. Lamella-resolved elastic restoring torque estimated from the analytical bending model.

Segment	r_i (μm)	F_i (mN)	$\tau_{e,i}=F_i r_i$ (nN m)	Cumulative τ_{cum}, (nN m)
L1	840	0.26	215	215
L2	580	0.54	312	527
L3	400	1.13	452	979
L4	360	1.40	502	1482
L5	260	2.68	696	2177

The outermost lamella therefore has an elastic restoring torque of approximately:

$$\tau_{e,L1} = 215\ nN\ m$$

whereas the cumulative elastic torque of the full modeled multilamellar structure is approximately:

$$\tau_{e,cum} = 2180nN\ m$$

These values provide the elastic-torque basis for comparison with the SQUID-derived magnetic torque. In the dual magneto-mechanical mechanism, magnetic torque initiates local release of the outer lamellae, while stored elastic strain energy contributes to cascaded unrolling and rapid recoil. A full FEA treatment of the coupled magnetic-contact-friction problem would be a valuable direction for future work.

SI 6. Magnetic–elastic torque comparison supporting the dual mechanism

To evaluate whether this magnetic torque is sufficient to overcome the elastic resistance of the scroll, it was compared with the elastic restoring torque obtained from the analytical bending model described in Section SI 5. For the mechanically opened 15 mm scroll, the restoring torque of the outermost lamella was:

$$\tau_{e,L1} = 215\text{nN m}$$

whereas the cumulative elastic torque of the full modeled multilamellar structure was:

$$\tau_{e,cum} = 2180\text{nN m}$$

Thus, the magnetic torque in the onset window is comparable to the restoring torque of the outermost lamella, but remains far below the cumulative elastic torque of the full scroll. This indicates that the magnetic field can initiate local bending and partially release the outer lamella, but cannot quasi-statically overcome the full elastic resistance of the multilamellar structure.

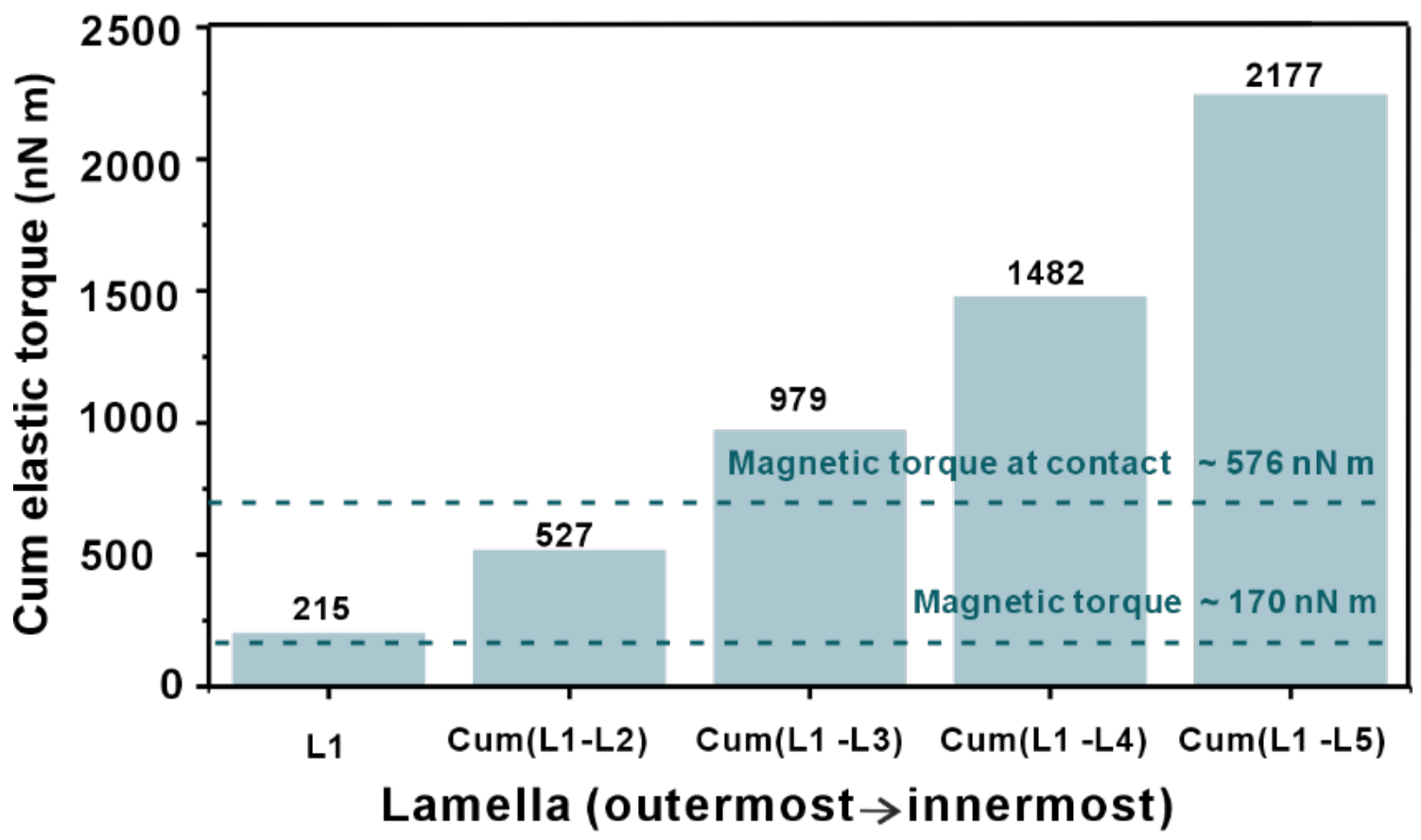

Figure S14. Comparison of cumulative elastic restoring torque and magnetic torque in the 15 mm VNF/Fe_3O_4 microscroll.

Bar plots show the cumulative elastic torque associated with progressive engagement of lamellar segments from the outermost layer (L1) to the full multilamellar structure (L1–L5), as obtained from the analytical bending model. Dashed horizontal lines indicate the representative magnetic torque during the actuation-onset regime (~170 nN m) and the maximum magnetic torque at direct magnet contact (~576 nN m). The comparison shows that the magnetic torque is sufficient to initiate opening of the outer lamellae, but remains far below the total elastic torque stored in the complete scroll.

To compare the magnetic and elastic contributions on a common basis, the SQUID-derived magnetic torque was evaluated against the elastic restoring torque obtained from the analytical bending model. Within the experimentally relevant onset window for actuation, corresponding magnet–scroll distance of approximately 5–7 mm, the magnetic torque is on the order of 10^2 nN m, whereas at direct magnet contact it reaches approximately 576 nN m. In comparison, the elastic restoring torque of the outermost lamella is approximately $\tau_{e,L1} \approx 215$ nN m, while the cumulative elastic torque of the full modeled multilamellar structure reaches $\tau_{e,total} \approx 2177$ nN m. Thus, the magnetic torque is sufficient to initiate local bending and partial release of the outer lamella and, at contact, to guide opening of the first several outer layers, but it remains well below the total elastic resistance stored in the tightly curved inner region. This comparison supports the proposed dual magneto-mechanical mechanism, in which the magnetic field acts as a trigger and directional guide, while stored elastic strain energy drives cascade-like unrolling and rapid recoil.

SI 7. Long-term cyclic stability and additional functional demonstrations

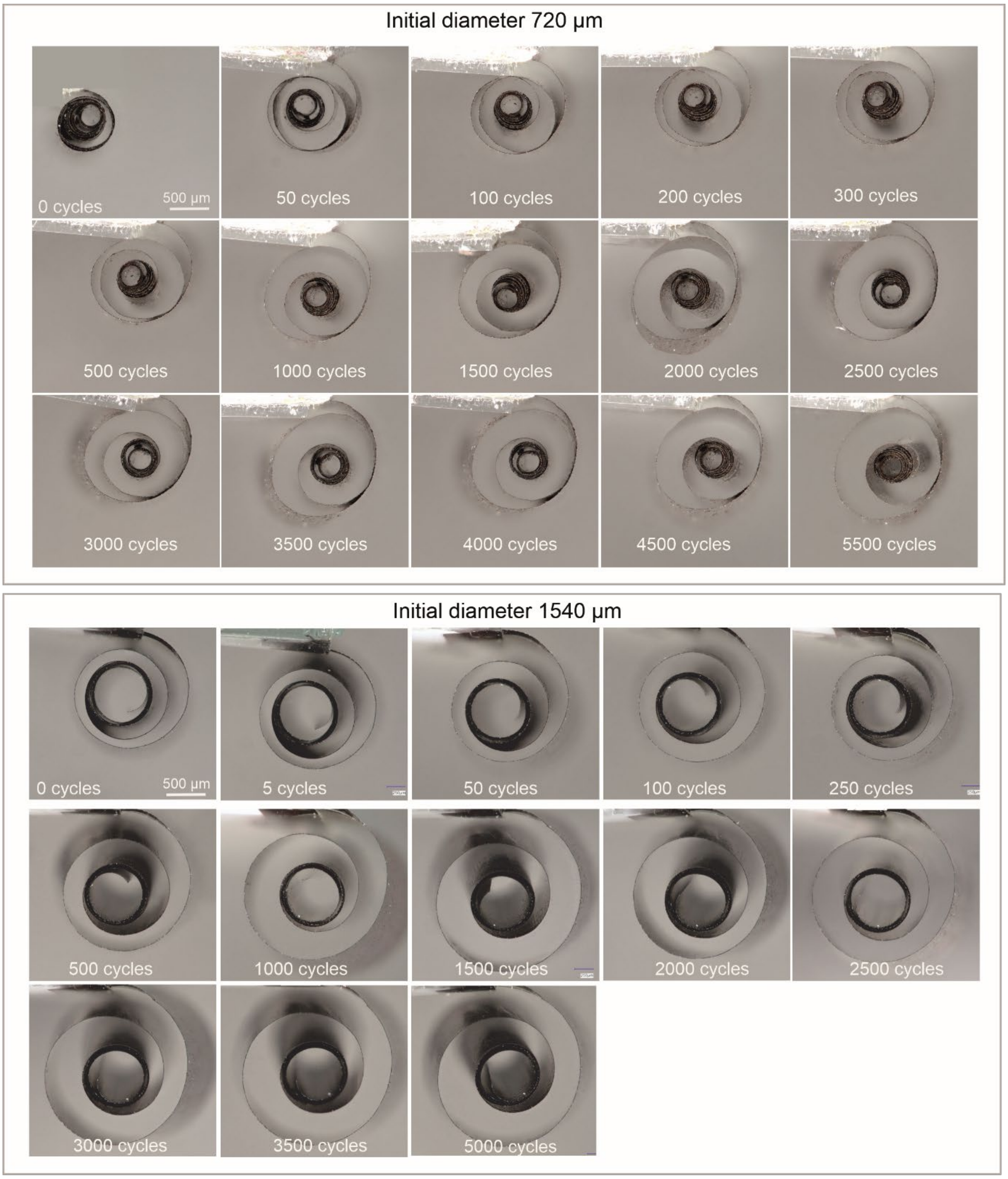

Figure S15. Cross-sectional optical images of the VNF/Fe_3O_4 microscrolls after selected magnetic actuation cycle numbers. The images show gradual relaxation of the outer lamellae while the compact inner core remains preserved, supporting the interpretation that cyclic actuation leads to limited geometric accommodation rather than structural failure.

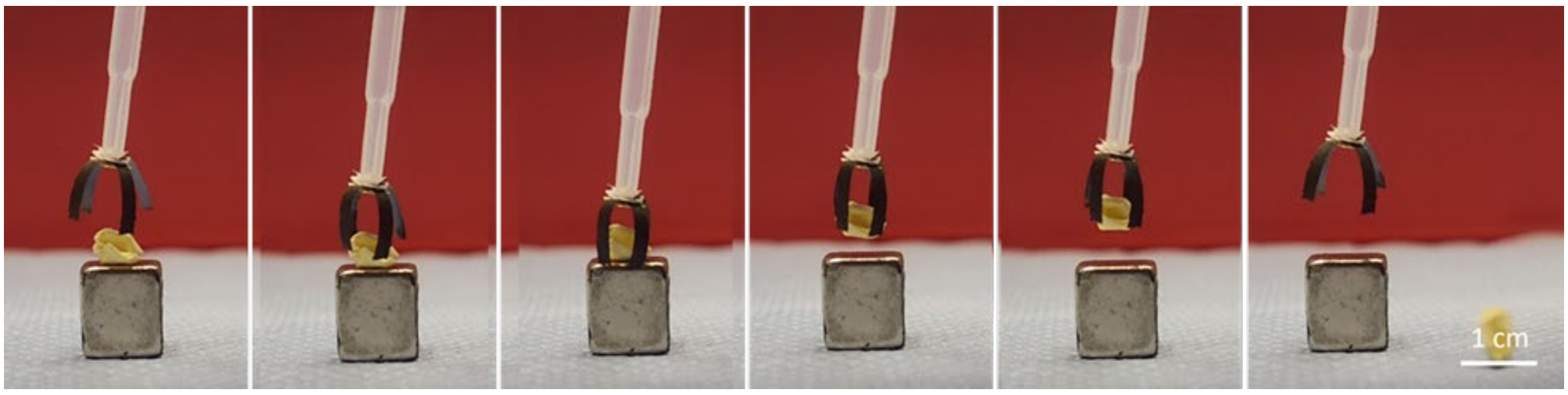

Figure S16. Gripping demonstration using four VNF/Fe_3O_4 hybrid-film strips. The strips were fabricated by reducing the scrolling angle to < 30°, producing semi-rolled actuator geometries capable of cooperative inward motion. When exposed to the magnetic field of a centrally positioned NdFeB magnet, all four strips simultaneously curled inward and grasped a lightweight paper payload (4 mg). Upon field removal, the strips elastically relaxed and released the object. This example illustrates how the same hybrid material system can be reconfigured into alternative actuator geometries beyond microscrolls, expanding the design space for untethered object manipulation.

SI 8. Performance metric calculations

This section describes the methodology used to calculate the performance metrics of the microscroll actuator (11 wt% Fe_3O_4 hybrid film thickness 4.2 µm). The tested scroll had a width of 2 mm, a scrolling length of 15 mm, and a mass of ~0.4 mg. All parameters were derived from experimental measurements during the lifting demonstration (Video S7), together with standard analytical expressions used for soft magnetic actuators.[1-3]

Mechanical work output

The mechanical work output was calculated using the classical expression:

$$W_{out} = m_t \cdot g \cdot \Delta y$$

where m_t = 13 mg is the lifted mass, g = 9.81m s^{-2} is gravitational acceleration, and Δy = 8 mm is the vertical lifting distance. This yields a mechanical work output of 1.02×10^{-6} J.

Magnetic input energy: Volume-based approximation

Quantifying input energy in magnetically actuated, untethered systems is inherently challenging because a permanent magnet does not supply energy in the classical sense. Instead, mechanical work arises from changes in magnetic potential energy as the actuator moves through the magnetic field. To obtain an upper-bound estimate, we approximate the accessible magnetic energy as the field energy stored within the volume of the hybrid film prior to scrolling:

$$V_{film} = 15\text{ mm} \cdot 2\text{ mm} \cdot 4.2\text{ µm} = 1.26 \times 10^{-10}\text{ m}^3$$

$$E_{in} = \frac{1}{2}\mu_0 H^2 V_{film}$$

where $H = \frac{B}{\mu_0}$ and with: $\mu^0 = 4\pi \cdot 10^{-7}\text{ Hm}^{-1}$

Using a representative local magnetic flux density of B = 60 mT:

This yields:

$$E_{in} \approx 1.81 \cdot 10^{-7}\text{ J}$$

This expression represents a field-volume estimate rather than the true mechanical work delivered by the magnet and therefore serves only as a comparison scale rather than a measure of actual energy delivery.

Apparent magneto-mechanical amplification factor

To avoid implying classical energy conversion efficiency, we report:

$$\Lambda = \frac{W_{mech,out}}{E_{mag}} \approx 5.6$$

This parameter is a comparison metric, not a thermodynamic efficiency. Values greater than 1 reflect the contribution of elastic strain energy stored during scroll formation, released cooperatively during actuation. Thus, the ratio captures dual-energy actuation, not thermodynamic efficiency.

Additional Performance Metrics

1. Unrolling ratio (geometric expansion)

$$\varepsilon = \left(\frac{\mathrm{L} - \mathrm{D}}{\mathrm{D}}\right) \cdot 100 = \left(\frac{15\ \mathrm{mm} - 1\ \mathrm{mm}}{1\ \mathrm{mm}}\right) \cdot 100 = 1400\%$$

2. Specific lifting performance

$$\mathrm{Ratio} = \frac{\mathrm{m_{load}}}{\mathrm{m_{actuator}}} = \frac{13\ \mathrm{mg}}{0.4\ \mathrm{mg}} = 32.5$$

3. Actuation stress

$$\sigma = \frac{\mathrm{F}}{\mathrm{A}} = \frac{\mathrm{m} \cdot \mathrm{g}}{\mathrm{W} \cdot \mathrm{t}} \approx 15.2\ \mathrm{kPa}$$

4. Specific work

$$\frac{\mathrm{W}}{\mathrm{m_{actuator}}} = \frac{1.02 \cdot 10^{-6}\ \mathrm{J}}{4 \cdot 10^{-7}\ \mathrm{kg}} = 2.55\ \mathrm{J\ kg^{-1}}$$

5. Work density (film-volume based)

$$\text{Work density} = \frac{W_{out}}{V_{film}} = \frac{1.02 \cdot 10^{-6}\ \mathrm{J}}{1.26 \cdot 10^{-10}\ \mathrm{m^3}} \approx 8.1\ \mathrm{kJ\ m^{-3}}$$

6. Power output

$$\frac{\mathrm{W}}{\text{cycle time}} = \frac{1.02\ \cdot 10^{-6}\ \mathrm{J}}{0.15\ \mathrm{s}}\ =\ 6.8\ \mu\mathrm{W}$$

7. Specific power

$$\frac{\text{Power}}{\mathrm{m_{actuator}}} = \frac{6.8\ \cdot 10^{-6}\ \mathrm{W}}{4\ \cdot 10^{-7}\ \mathrm{kg}}\ =\ 17\ \mathrm{Wkg^{-1}}$$

8. Frequency

$$\mathrm{f}\ = \frac{1}{\text{cycle time}} = \frac{1}{0.15\ \mathrm{s}} =\ 6.67\ \mathrm{Hz}$$

9. Footprint reduction

$$\text{Reduction}\ = (1 - \frac{\mathrm{A_{rolled}}}{\mathrm{A_{open}}})\ \cdot\ 100 = (1 - \frac{2\ \mathrm{mm}^2}{30\ \mathrm{mm}^2})\cdot\ 100\ =\ 93\%$$

Longer scrolls (20–25 mm) reach up to 96% reduction due to larger diameters.

Methodological Notes

The estimation of magnetic input energy remains a key challenge in passive, field-driven magnetic actuators. Depending on whether the reference volume is taken as the rolled geometry, a hollow cylindrical shell, or the unrolled film volume, the apparent return ratio can vary substantially. The film-volume basis used here provides a consistent material-normalized comparison and avoids assumptions about scroll morphology. These values should therefore be interpreted as indicative measures of magneto-mechanical coupling rather than strict energy-conversion efficiencies. More refined models incorporating magnetic hysteresis, field gradients, and time-resolved torque profiles are needed for full quantification.

Video Documentation

Demonstration videos (Videos S4–S8) were recorded using a mobile phone camera and were used solely for qualitative visualization of actuator motion. These recordings served to illustrate overall deformation modes, gripping behavior, and unrolling dynamics. All quantitative measurements, including force, displacement, strain, and actuation timing, were obtained exclusively using calibrated optical microscopy systems and precision mechanical testing instruments.

SI 9. Comparative benchmarking with reported actuator systems

Table S4
Reported values are representative literature values and may depend on experimental configuration, normalization methods, and actuator geometry.

Parameter	Sol et al. 2019 [4]	Lin & Liu 2019 [5]	Liu et al. 2019 [6]	Dezaki & Bodaghi 2023 [7]	This work
Material system	Liquid crystal network (LCN)	Electroactive polymer spiral actuator	Shape-memory polymer (DiAPLEX/IROGRAN) + Fe microparticles	Magneto-active soft composite	V_2O_5 nanofiber / Fe_3O_4 ceramic hybrid
Structural concept	Tapered scroll actuator	Proboscis-inspired spiral strip	Programmable magnetic scrolls and snappers	Prestressed spiral soft actuator	Multilamellar ceramic microscroll
Mechanical strategy	Anisotropic thermomechanical strain gradient	Electrically induced spiral winding	Magnetic + photothermal reconfiguration	Prestress-driven spiral deformation	Magnetic torque + elastic recoil release
Actuation mechanism	Photothermal bending/coiling	Electrothermal spiral contraction	Magnetically assisted SMP softening and reconfiguration	Magnetic deformation of prestressed elastomer	Dual magneto-mechanical actuation
Stimulus	Heat and blue light (455 nm)	Electrical current	Magnetic field + NIR light	Magnetic field	Magnetic field
Typical magnetic field	—	—	~57 mT	~100–300 mT	~60 mT
Response time	Minutes	Seconds	3–21 s	~0.5–2 s	<150 ms
Structural rigidity	Soft polymeric	Soft polymeric	Soft SMP	Highly compliant elastomer	Rigid ceramic multilamellar hybrid
Programmability	Moderate	Moderate	Very high (reconfigurable shapes, bistability, locking)	Moderate	Very high (geometry-encoded scrolling)
Lifting capability	Not reported	Not reported	~20× own weight	Limited	>30× own weight
Cycling stability	Reversible actuation (cycles not quantified)	Not reported	Multiple reversible cycles	Reversible deformation	>5000 cycles
Scale	mm–cm	cm	cm	cm	µm–mm
Fabrication complexity	Moderate	Moderate	High (SMP processing + photothermal control)	Moderate	Simple razor-blade scrolling
Key feature	Light-driven tapered scroll motion	Bioinspired spiral contraction	Reconfigurable magnetic scrolls/snappers with locking	Prestressed magnetic spiral actuation	Ultrafast ceramic magneto-elastic recoil scrolling

Table S5

Material class	Structural concept	Magnetic stimulus	Representative actuation metric	Representative actuation behavior	Operational repeatability	Scale	Key feature	Reference
Ceramic-based magnetic nanofiber hybrid film	Razor-blade mechanically scrolled multilamellar magnetic microscrolls	Uniform magnetic field (~60 mT)	Work density ≈8.1 kJ m^{-3}; response <150 ms; lifts >30× own weight	Fast reversible opening/unrolling of scroll architecture	>5000 cycles	Microscale scrolls	Dual magneto-mechanical actuation (magnetic torque + stored elastic energy release)	This work –
PDMS magnetic composite + planar coils	Near-field programmable soft actuator arrays	Electromagnetic near-field (~2 mT; gradients ~1 T m^{-1})	Work density ≈0.3 kJ m^{-3} A^{-1}; 25 Hz	Lifting, tilting, grasping, pulling	Repeated high-frequency operation	mm-scale	Independently addressable ultrathin actuator arrays	Richter et al., 2023 [8]
Fiber-based magnetic elastomer	Strain-engineered helical fibers	Oscillating magnetic field (23–45 mT)	Locomotion ≈2–3 mm s^{-1}	Crawling and walking robots	Reversible cyclic locomotion	Fiber/millimeter scale	High-throughput scalable fiber robots	Lee et al., 2023 [9]
Magnetic shape-memory polymer composite	Reconfigurable magnetic origami	Magnetic field + photothermal activation	Folding up to 180°	Sequential programmable folding	Reversible folding cycles	Thin-film/cm scale	On-board sensing + magnetic cognition	Ha et al., 2021 [10]
Hard-magnetic elastomer	Origami-inspired folded architectures	External magnetic field (0–90 mT)	Body contraction to 65%; response <0.5 s	2D-to-3D morphing, rolling locomotion	Reversible deformation	mm–cm scale	Programmable origami magnetization	Tang et al., 2021 [11]
PDMS/NdFeB magnetic soft composite	Microstructure-enhanced segmented actuator	Electromagnetic actuation	Crawling speed ≈35 mm s^{-1}	Inchworm and quadruped locomotion	Stable repeated motion	20 × 20 mm films	High force + low stiffness simultaneously	Zhu et al., 2025 [12]
Reprogrammable magnetic elastomer pixels	Pixel-assembled magnetic soft actuator	Homogeneous magnetic field (5–25 mT)	Swimming ≈6.6 mm s^{-1}	Reconfigurable morphing/swimming/gripping	50 reuse cycles	mm-scale	Reprogrammable magnetic pixel assembly	Zhao et al., 2024 [13]
Fe_3O_4/cellulose paper composite	Magnetic origami-like paper actuator	Permanent magnetic field (0–230 mT)	100% strain; ≈10 mm deflection	Folding, gripping, accordion deformation	Stable over 100 cycles	Paper strips/cm scale	Lightweight biodegradable magnetic paper actuator	Wang et al., 2018 [14]

The selected references in Table S5 represent distinct and widely recognized design strategies in the field of magnetic soft actuators, including classical magnetic bending films, reconfigurable magnetic origami, strain-programmed helical architectures, and low-field microscale magnetic actuation systems. These studies were specifically chosen because they provide structurally and conceptually relevant comparisons to the present VNF–Fe_3O_4 microscrolls in terms of untethered magnetic actuation, architected deformation, and programmable shape transformation. Compared with representative polymeric and paper-based magnetic actuator systems, our ceramic nanofiber-based microscrolls combine rapid magneto-elastic opening, compact-to-open geometrical transformation, high cyclic durability, and significant load-lifting capability under relatively low magnetic fields.

References

1. M. Li, A. Pal, A. Aghakhani, A. P. Francesch, M. Sitti, "Soft Actuators for Real-World Applications," *Nature Reviews Materials* 7 (2022): 235. https://doi.org/10.1038/s41578-021-00389-7
2. J.-P. Yonnet, C. Chillet, H. Allag, L. Chouikhi, "Analytical Calculation of the Energy in a Permanent Magnet," Journal of Modern Physics 7, no. 16 (2016): 2281. http://dx.doi.org/10.4236/jmp.2016.716196
3. L. Shui, L. Zhu, Z. Yang, Y. Liu, X. Chen, "Energy Efficiency of Mobile Soft Robots," Soft Matter 13, no. 44 (2017): 8223. https://doi.org/10.1039/c7sm01617d
4. J. A. H. P. Sol, A. R. Peeketi, N. Vyas, A. P. H. J. Schenning, R. K. Annabattula, M. G. Debije, "Butterfly proboscis-inspired tight rolling tapered soft actuators," *Chemical Communications* 55, no. 12 (2019): 1726. https://doi.org/10.1039/c8cc09915d
5. P.-W. Lin, C.-H. Liu, "Bio-Inspired Soft Proboscis Actuator Driven by Dielectric Elastomer Fluid Transducers," *Polymers* 11, no. 1 (2019): 142. https://doi.org/10.3390/polym11010142
6. J. A.-C. Liu, J. H. Gillen, S. R. Mishra, E. E. Evans, J. B. Tracy, "Photothermally and magnetically controlled reconfiguration of polymer coposites for soft robotics," *Science Advances* 5, no. 8 (2019): eaaw2897. https://doi.org/10.1126/sciadv.aaw2897
7. M. L. Dezaki, M. Bodaghi, "Magnetically Controlled Bio-Inspired Elastomeric Actuators with High Mechanical Energy Storage," *Soft Matter* 19, no. 16 (2023): 3015. https://doi.org/10.1039/d3sm00266g
8. M. Richter, J. Sikorski, P. Makushko, et al., "Locally Addressable Energy Efficient Actuation of Magnetic Soft Actuator Array Systems," *Advanced Science* 10, no. 24 (2023): 2302077. https://doi.org/10.1002/advs.202302077

9. Y. Lee, F. Koehler, T. Dillon, G. Loke, Y. Kim, J. Marion, et al., "Magnetically Actuated Fiber-Based Soft Robots," *Advanced Materials* 35, no. 38 (2023): 2301916. https://doi.org/10.1002/adma.202301916
10. M. Ha, G. S. C. Bermudez, J. A.-C. Liu, et al., "Reconfigurable Magnetic Origami Actuators with On-Board Sensing for Guided Assembly," *Advanced Materials* 33, no. 25 (2021): 2008751. https://doi.org/10.1002/adma.202008751
11. D. Tang, C. Zhang, H. Sun, et al., "Origami-Inspired Magnetic-Driven Soft Actuators with Programmable Designs and Multiple Applications," *Nano Energy* 89, no. B (2021): 106424. https://doi.org/10.1016/j.nanoen.2021.106424
12. H. Zhu, W. Song, H. Tian, et al., "Microstructure-Enhanced Magnetic-Driven Soft Actuator with High Force and Large Deformation," *ACS Applied Materials & Interfaces* 17, no. 39 (2025): 55463. https://doi.org/10.1021/acsami.5c14850
13. X. Zhao, H. Yao, Y. Lv, et al., "Reprogrammable Magnetic Soft Actuators with Microfluidic Functional Modules via Pixel-Assembly," *Small* 20, no. 28 (2024): 2310009. https://doi.org/10.1002/smll.202310009
14. X. Wang, B. Han, R.-P. Yu, et al., "Magnetic-responsive Fe_3O_4 nanoparticle-impregnated cellulose paper actuators," *Extreme Mechanics Letters* 25 (2018): 53. https://doi.org/10.1016/j.eml.2018.10.003

Video S1.

Scroll fabrication recorded under digital light microscopy, showing sequential delamination and winding formation as the hybrid film rolls into a microscroll under a fixed razor blade.

Video S2.

Multidirectional actuation of VNF/ Fe_3O_4 microscrolls using four permanent magnets. Scrolls respond to magnetic fields applied from three different directions within a 180° actuation range including simultaneous actuation of several scrolls (20 mm length, 2 mm width).

Video S3.

Side-view recording of the opening and closing process of a microscroll during magnetic actuation, highlighting partial unrolling, cascaded propagation, and elastic recoil.

Video S4

Optical-microscopy recording of the unrolling and re-rolling cycle of a microscroll during magnetic actuation, showing lamellar motion and overall deformation dynamics. The recording is played back at 2 times its real-time speed.

Video S5.

Mechanical opening of a microscroll using a fixed carbon rod inserted into the scroll center. The recording is played back at 120 times its real-time speed.

Video S6.

Opening and closing of a compact VNF/Fe_3O_4 microscroll after 5000 magnetic actuation cycles. The video shows reversible magnetically induced unrolling and elastic re-rolling of a 20 mm-long microscroll with an initial outer diameter of ~720 µm, demonstrating retained actuation capability after extended cyclic operation.

Video S7.

A 0.4 mg scroll (15 mm length, 2 mm width) lifting a 13 mg wooden rod, corresponding to a lifting ratio of ~32.5× its own mass.

Video S8.

A microscroll (20 mm length, 2 mm width) lifting and lowering a 4 mg carbon rod to close and open an electrical circuit, functioning as a fully untethered magnetic switch.
